# Targeting transcriptional regulation of SARS-CoV-2 entry factors *ACE2* and *TMPRSS2*

**DOI:** 10.1073/pnas.2021450118

**Published:** 2020-12-28

**Authors:** Yuanyuan Qiao, Xiao-Ming Wang, Rahul Mannan, Sethuramasundaram Pitchiaya, Yuping Zhang, Jesse W. Wotring, Lanbo Xiao, Dan R. Robinson, Yi-Mi Wu, Jean Ching-Yi Tien, Xuhong Cao, Stephanie A. Simko, Ingrid J. Apel, Pushpinder Bawa, Steven Kregel, Sathiya P. Narayanan, Gregory Raskind, Stephanie J. Ellison, Abhijit Parolia, Sylvia Zelenka-Wang, Lisa McMurry, Fengyun Su, Rui Wang, Yunhui Cheng, Andrew D. Delekta, Zejie Mei, Carla D. Pretto, Shaomeng Wang, Rohit Mehra, Jonathan Z. Sexton, Arul M. Chinnaiyan

**Affiliations:** ^a^Michigan Center for Translational Pathology, University of Michigan, Ann Arbor, MI 48109;; ^b^Department of Pathology, University of Michigan, Ann Arbor, MI 48109;; ^c^Rogel Cancer Center, University of Michigan, Ann Arbor, MI 48109;; ^d^Department of Medicinal Chemistry, College of Pharmacy, University of Michigan, Ann Arbor, MI 48109;; ^e^Howard Hughes Medical Institute, University of Michigan, Ann Arbor, MI 48109;; ^f^State Key Laboratory of Cell Biology, CAS Center for Excellence in Molecular Cell Science, University of Chinese Academy of Sciences, Shanghai 200031, China;; ^g^Department of Internal Medicine, University of Michigan, Ann Arbor, MI 48109;; ^h^Department of Pharmacology, University of Michigan, Ann Arbor, MI 48109;; ^i^Center for Drug Repurposing, University of Michigan, Ann Arbor, MI 48109;; ^j^Michigan Institute for Clinical and Health Research, University of Michigan, Ann Arbor, MI 48109;; ^k^Department of Urology, University of Michigan, Ann Arbor, MI 48109

**Keywords:** SARS-CoV-2, TMPRSS2, androgen receptor, BET inhibitors, ACE2

## Abstract

Severe acute respiratory syndrome coronavirus 2 (SARS-CoV-2), the virus responsible for COVID-19, employs two key host proteins to gain entry and replicate within cells, angiotensin-converting enzyme 2 (ACE2) and the cell surface transmembrane protease serine 2 (TMPRSS2). *TMPRSS2* was first characterized as an androgen-regulated gene in the prostate. Supporting a role for sex hormones, males relative to females are disproportionately affected by COVID-19 in terms of mortality and morbidity. Several studies, including one employing a large epidemiological cohort, suggested that blocking androgen signaling is protective against COVID-19. Here, we demonstrate that androgens regulate the expression of *ACE2*, *TMPRSS2*, and androgen receptor (AR) in subsets of lung epithelial cells. AR levels are markedly elevated in males relative to females greater than 70 y of age. In males greater than 70 y old, smoking was associated with elevated levels of AR and ACE2 in lung epithelial cells. Transcriptional repression of the AR enhanceosome with AR or bromodomain and extraterminal domain (BET) antagonists inhibited SARS-CoV-2 infection in vitro. Taken together, these studies support further investigation of transcriptional inhibition of critical host factors in the treatment or prevention of COVID-19.

## Significance

New therapeutic targets are urgently needed against SARS-CoV-2, the coronavirus responsible for the COVID-19 pandemic. Results in this study show that targeting the transcriptional regulation of host entry factors *TMPRSS2* and *ACE2* is a viable treatment strategy to prevent SARS-CoV-2 infection. In particular, inhibitors of androgen receptor (AR) or bromodomain and extraterminal domain (BET) proteins are effective against SARS-CoV-2 infection. AR inhibitors are already approved in the clinic for treatment of prostate cancer and are under investigation in COVID-19 patients; BET inhibitors are also in clinical development for other indications and could be rapidly repurposed for COVID-19.

The COVID-19 pandemic has become one of the greatest public health challenges in modern times, leading to an unprecedented surge in research efforts to develop vaccines and treatments (1). The genome of severe acute respiratory syndrome coronavirus 2 (SARS-CoV-2), the coronavirus responsible for COVID-19, is a positive, single-stranded RNA that encodes nonstructural and structural proteins required for the viral life cycle. Among these are its four main structural proteins: N, Nucleocapsid; E, Envelope; M, Membrane; and S, Spike (2). The Spike glycoprotein plays a pivotal role in SARS-CoV-2 infection by recognizing and attaching to the angiotensin-converting enzyme 2 (ACE2) transmembrane protein on host cells (3). The Spike protein is also cleaved and activated by cell surface transmembrane protease serine 2 (TMPRSS2) to facilitate membrane fusion and entry (4).

Targeting the expression or activity of these host receptors has confirmed their critical role in the pathogenicity of coronavirus infections (4–6). Studies with *TMPRSS2* transgenic knockout mice have shown that loss of *TMPRSS2* can reduce coronavirus replication in lungs, elicit a weaker proinflammatory response, and result in a milder lung pathology (5). SARS-CoV-2 entry into cells is also decreased upon TMPRSS2 functional inhibition by the serine protease inhibitor camostat (4). Likewise, ACE2 antibodies or soluble recombinant ACE2 can attenuate viral entry and infection by SARS-CoV-2 (4, 6). Thus, a better understanding of regulatory mechanisms that control expression levels of ACE2 and TMPRSS2 could be key to developing effective novel treatments for SARS-CoV-2 infections. Interestingly, TMPRSS2 has been widely studied in the context of prostate cancer, where it is highly expressed, and *TMPRSS2* expression is increased in response to androgens through direct transcriptional regulation by the androgen receptor (AR) (7). Oncogenic androgen-regulated *TMPRSS2-ETS* gene fusions are also found in upward of 50% of prostate cancers (8, 9).

Since the earliest demographics data were emerging from the COVID-19 pandemic, it became clear that there is a gender disparity in severity of disease course which persists across nations, with males having higher hospitalization and mortality rates than females (10, 11). The reasons for these gender disparities may be multifactorial, but one possible explanation could be differences in levels of sex hormones, such as androgens, and the transcriptional signaling networks that subsequently occur in males versus females, including up-regulation of the *TMPRSS2* host entry factor in males. This has raised the hypothesis that inhibition of AR activity and down-regulation of *TMPRSS2* may prevent SARS-CoV-2 infection (12). In support of this theory, a retrospective study in Italy analyzing rates of SARS-CoV-2 infectivity among prostate cancer patients found a significantly reduced incidence in patients receiving androgen deprivation therapy (ADT) (13). Similarly, a small prospective study of patients hospitalized due to COVID-19 observed a decreased rate of intensive care unit admissions among men that had been taking antiandrogens for at least 6 mo prior to hospitalization (14). Conversely, another large prospective study reported no difference in risk of SARS-CoV-2 infection with ADT in prostate cancer patients, suggesting the need for further research into the role of androgens in regulating viral entry factors and disease course (15). Additionally, the interplay of androgens with other variables, such as comorbid health conditions, age, and smoking, remains to be fully elucidated, with initial evidence suggesting a correlation between current smoking status, *ACE2*/*TMPRSS2* expression, and AR signaling (10, 16).

Given these knowledge gaps, the goals of the current study were to determine which cells of the upper airway tract express ACE2 and TMPRSS2 and test whether their expressions could be therapeutically targeted by AR inhibitors used in prostate cancer treatment. Coexpression of SARS-CoV-2 host entry factors and AR was observed in alveolar and bronchial epithelial cells, with significantly higher levels of ACE2 and AR in the lungs of aged male smokers. Importantly, *TMPRSS2* and *ACE2* expressions were decreased with therapies that directly target AR, as well as inhibitors of bromodomain and extraterminal domain (BET) proteins, known epigenetic regulators of AR transcriptional activity (17). Critically, these therapies led to decreased SARS-CoV-2 infection in cellular models, and, thus, these findings support further studies into AR and BET inhibitors as candidate treatment modalities for COVID-19.

## Results

### Single-Cell Sequencing Analysis of *AR*, *ACE2*, and *TMPRSS2* Expression in Lungs and Their Responses to Androgen.

To determine whether androgen signaling regulates the expression of SARS-CoV-2 entry factors *ACE2* and *TMPRSS2*, we used several human cell lines that are reported to be permissive to infection with either pseudotyped virus expressing SARS-CoV-2 Spike protein or isolated SARS-CoV-2 (4, 18). Despite being well characterized as suitable cell lines for antiviral drug screens with SARS-CoV-2, lung adenocarcinoma Calu-3 and colon adenocarcinoma Caco-2 cell lines both lack robust endogenous AR expression, rendering screening results uninterpretable for AR-targeting drugs. Here, we confirmed that neither androgen stimulation nor AR antagonists altered the levels of *TMPRSS2* and *ACE2* in Calu-3 and Caco-2 cells (*SI Appendix*, Fig. S1 *A* and *B*). On the other hand, we discovered that the BET inhibitor JQ1 and degrader ZBC260 consistently down-regulated *ACE2* in Calu-3 (*SI Appendix*, Fig. S1*A*) and *TMPRSS2* and *ACE2* in Caco-2 cells (*SI Appendix*, Fig. S1*B*), suggesting that BET proteins may play a role in regulating SARS-CoV-2 entry factor expression. Additionally, in other cell lines originating from the lung lineage with high endogenous expression of *AR*, *TMPRSS2*, and *ACE2*, we found that both androgen and AR antagonists also did not change *TMPRSS2* and *ACE2* messenger RNA (mRNA) levels in bulk gene expression analysis (*SI Appendix*, Fig. S1 *C*–*F*). The varying endogenous expressions of *AR*, *TMPRSS2*, and *ACE2* in lung cell lines limit their use in SARS-CoV-2 research; thus, there is a need for understanding their expression patterns in the lung at the single-cell level.

**Fig. 1.**
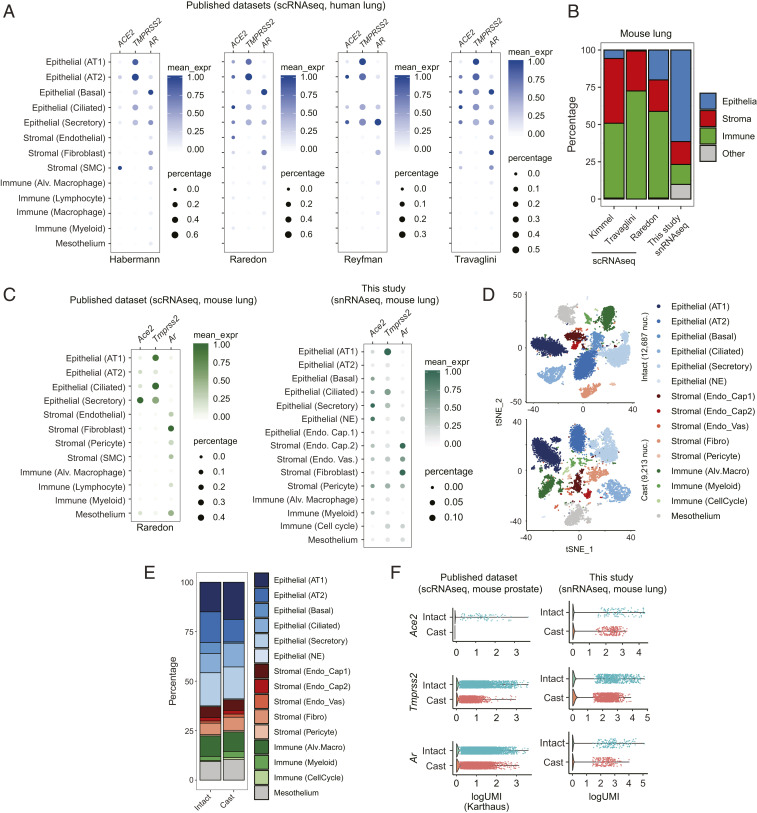
Single-cell analysis of host SARS-CoV-2 entry factors and AR in human and mouse lungs. (*A*) Bubble plots of *ACE2*, *TMPRSS2*, and *AR* expression from publicly available scRNAseq datasets of human lung. Color bar represents mean expression (mean_expr) of each gene in specific cell types, and bubble size represents the percentage of cells in each cell type that expresses that gene (here and in *C*). Each plot is labeled below with an identifier, reflecting the name of the first author of the appropriate manuscript (same with *C*) (20−23). AT1, alveolar type 1; AT2, alveolar type 2; SMC, smooth muscle cell; Alv., alveolar. (*B*) Stacked bar plot representing the fraction of appropriate, color-coded cell types obtained by scRNAseq (public dataset) and snRNAseq (this study) of mouse lung. (*C*) Bubble plot of *Ace2*, *Tmprss2,* and *Ar* expression from publicly available scRNAseq dataset and snRNAseq dataset from this study of mouse lung. NE, neuroendocrine; Endo. Cap., endothelial capillary; Endo. Vas., endothelial vasculature. (*D*) The t-distributed stochastic neighbor embedding (tSNE) plots of snRNAseq data from mouse lungs collected from intact or castrated (cast) mice. Each dot represents a cell, and each cell type identified by our analysis is distinctly color coded. Fibro, fibroblast. (*E*) Stacked bar plot representing the fraction of appropriate, color-coded cell types obtained by snRNAseq of lungs from intact and castrated (cast) mice. (*F*) Combined violin and scatter plots representing the expression of *Ace2*, *Tmprss2,* and *A*r in lungs from intact and castrated mice from publicly available datasets of mouse prostate and this study of mouse lung. Each dot is an individual cell (only nonzero values are indicated), and all cell types from each sample have been included here to perform a pseudobulk gene expression analysis.

Given the complexity of the lungs, which comprise more than 25 distinct cell types including bronchial and alveolar cells (19–23), identification of specific cells that express *AR*, *TMPRSS2*, and *ACE2* genes will be critical to understanding the biology of SARS-CoV-2 infection. Thus, we performed bioinformatics analysis of published single-cell RNA sequencing (scRNAseq) data of human and murine lungs (19–23). The results demonstrated that *AR* was expressed with *TMPRSS2* and *ACE2* in several types of human (Fig. 1*A*) and murine (Fig. 1 *B* and *C* and *SI Appendix*, Fig. S2) lung epithelial cells, including alveolar (AT1 and AT2) epithelial cells, with maximal expression in ciliated and secretory epithelial cells (bronchial cells). This suggested that pulmonary *TMPRSS2* and *ACE2* in alveolar and bronchial cells had the potential to be regulated by AR.

To test this hypothesis by single-cell sequencing, we castrated adult immune-competent C57BL/6 male mice to create an androgen-deprived condition to compare lung tissue from intact and castrated mice. However, scRNAseq approaches require the dissociation of viable cells from the tissue milieu, and this method yielded suboptimal levels of lung epithelial cells. As an alternative strategy not affected by dissociation-induced artifacts (24), we performed single-nucleus RNA sequencing (snRNAseq) from frozen samples and found significant enrichment of lung epithelia (Fig. 1*B*). In fact, snRNAseq identified more cell types, including two additional epithelial cell types (basal and rare neuroendocrine/NE cells), and retained similar expression patterns of *Ar*, *Ace2*, and *Tmprss2* as compared to scRNAseq (Fig. 1*C*). Using this approach, we performed snRNAseq of lungs from intact and castrated mice and found no change in cellular composition between conditions (Fig. 1 *D* and *E*). Importantly, a global reduction of *Ace2*, *Tmprss2*, and *Ar* expression in lungs from the castrated sample was observed (Fig. 1*F*). Analysis of publicly available scRNAseq data of intact and castrated prostates exhibited similar regulation (Fig. 1*F*). Together, these data demonstrate that an androgen-deprived environment leads to decreased *Tmprss2* and *Ace2* expression in lung epithelial cells.

To compare expression levels of *AR*, *TMPRSS2*, and *ACE2* between lung and prostate, we analyzed publicly available datasets and calculated relative expressions by normalizing to a set of genes which were stably expressed across tissue lineages (20–23, 25, 26). In cells with detectable *TMPRSS2*, the expression levels were generally lower in human lung epithelial than prostate epithelial cells (*SI Appendix*, Fig. S3 *A* and *B*); however, in mice, *Tmprss2* was expressed at comparable levels in AT1 (alveolar) and ciliated cells (bronchial) of lung tissues with prostate luminal cells (*SI Appendix*, Fig. S3 *C* and *D*). *ACE2* detection was sparse in both lungs and prostates from humans and mice, but, noticeably, outlier expression of this gene was higher in lung AT2 cells compared to other cells in the lung and epithelial cells in the prostate of both humans and mice. *AR* detection was low in both human lung and prostate, whereas it was higher in mouse prostate epithelial cells compared to mouse lung epithelial cells. This analysis indicates that *TMPRSS2* and *ACE2* are expressed in both lung and prostate tissues in humans and mice, with higher relative *TMPRSS2* expression in human prostate epithelial cells.

### Androgen Positively Regulates Expression of AR and SARS-CoV-2 Entry Factors *Tmprss2* and *Ace2* in Murine Lungs.

To evaluate the effect of systemic androgen levels on murine lungs, we castrated adult immune-competent C57BL/6 male mice, and a group of castrated male mice were restimulated with testosterone for 5 d. To also test whether lungs from female mice are responsive to androgen stimulation, we treated intact female mice with testosterone (*SI Appendix*, Fig. S4*A*). Systemic testosterone levels from mouse serum confirmed down-regulation of testosterone upon castration in male mice and significant up-regulation by testosterone treatment in both castrated male and intact female mice (*SI Appendix*, Fig. S4*B*).

**Fig. 2.**
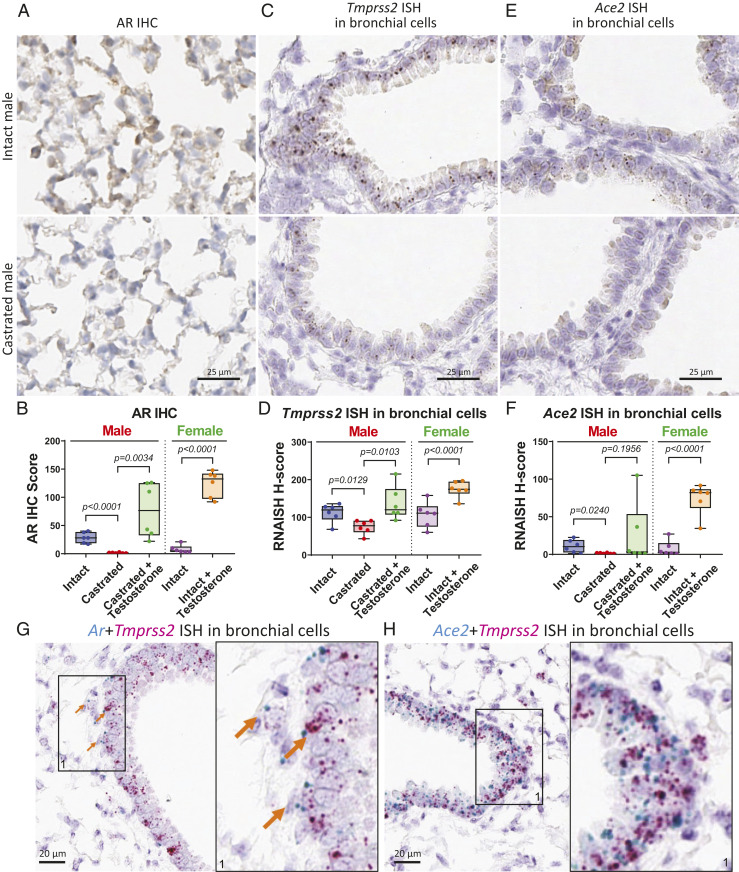
Expression levels of AR, *Tmprss2*, and *Ace2* in murine lungs are regulated by androgen. (*A*) Representative images of AR IHC in murine lungs from intact and castrated male mice. (*B*) Quantitative analysis of AR IHC score of murine lungs from male (intact, castrated, castrated + testosterone) and female (intact, intact + testosterone); *n* = 6 in each group, and *P* values were calculated by unpaired *t* test (here and in *D* and *F*). (*C*) Representative images of *Tmprss2* ISH and (*E*) *Ace2* ISH in murine bronchial cells from intact and castrated male mice. (*D*) Quantitative analysis of *Tmprss2* ISH and (*F*) *Ace2* ISH H-score in murine bronchial cells from male and female. (*G*) Representative image showing coexpression of *Ar* and *Tmprss2* ISH in murine bronchial cells. Arrows indicate cells expressing both *Ar* and *Tmprss2*. A magnified area (1) is shown on the right for this and panel *H*. (*H*) Representative image showing coexpression of *Ace2* and *Tmprss2* ISH in murine bronchial cells.

Levels of *Ar*, *Tmprss2*, and *Ace2* were examined using bulk lung tissue harvested from individual mice. The results showed no significant differences for *Ar* levels in treatment groups of male mice but a significant up-regulation in female mice treated with testosterone; however, *Tmprss2* and *Ace2* were not significantly altered between treatment groups in this bulk pooled tissue analysis (*SI Appendix*, Fig. S4*C*). Similar to mRNA levels, protein expression of ACE2 was not different among groups of pooled samples (*SI Appendix*, Fig. S4*D*). We further tested ACE2 protein expression from individual lung samples obtained from intact and castrated severe combined immunodeficiency male mice but again observed no difference (*SI Appendix*, Fig. S4*E*). However, given the coexpression patterns of *Ar*, *Tmprss2*, and *Ace2* at the single-cell level in lung and their regulation by castration (Fig. 1), we developed in situ analysis methods to analyze AR, *Tmprss2*, and *Ace2* at the cellular level in lung sections generated from C57BL/6 male (intact, castrated, castrated + testosterone) and female mice (intact, intact + testosterone). In murine lungs, AR was detected by immunohistochemistry (IHC) in nuclei, with localization in both bronchial and nonbronchial epithelial cells (Fig. 2*A* and *SI Appendix*, Fig. S5*A*). AR protein levels were calculated as an AR IHC score by factoring in IHC staining intensity and subcellular localization of AR protein. The AR IHC score was significantly lower in lungs of castrated male mice compared to intact male mice without changing subcellular localization of AR protein (Fig. 2 *A* and *B*). Baseline AR expression in lungs of females was notably lower than intact males. When mice were challenged with testosterone, AR IHC scores increased in lungs of castrated male mice and intact female mice (Fig. 2 *A* and *B* and *SI Appendix*, Fig. S5*A*), suggesting that pulmonary AR expression in bronchial and nonbronchial cells is positively regulated by androgen.

*Tmprss2* and *Ace2* were analyzed using lung sections of the same cohort. Consistent with single-cell sequencing analysis, *Tmprss2* RNA visualized by *Tmprss2* in situ hybridization (ISH) localized in both bronchial and nonbronchial cells (Fig. 2*C* and *SI Appendix*, Fig. S5 *A* and *B*) of murine lungs. Similar to AR protein, *Tmprss2* decreased in male bronchial cells upon castration and reactivated with testosterone treatment (Fig. 2 *C* and *D*). Similar up-regulation of *Tmprss2* ISH was also observed in intact female bronchial cells after testosterone treatment (Fig. 2*D* and *SI Appendix*, Fig. S5*A*). *Tmprss2* RNA in nonbronchial cells was shown to have the same regulation by androgen as in bronchial cells (*SI Appendix*, Fig. S5 *B* and *C*). However, *Ace2* RNA was only detectable in bronchial cells of murine lung. Bronchial expression of *Ace2* was down-regulated upon castration in male mice and significantly reactivated with testosterone stimulation in castrated male and intact female mice (Fig. 2 *E* and *F* and *SI Appendix*, Fig. S5*A*). To investigate ACE2 protein expression in lungs, we optimized ACE2 IHC to compare with *Ace2* ISH staining; lack of a suitable antibody for TMPRSS2 precluded its evaluation by IHC. Consistent with it being a known transmembrane protein, ACE2 IHC showed strong positive membrane staining in both bronchial and nonbronchial cells. This suggests that *Ace2* RNA may have a short half-life, or the *Ace2* ISH signal is too low to be detected in nonbronchial cells. Since almost all bronchial cells showed positive ACE2 IHC staining, quantitative analysis of ACE2 positive cells in nonbronchial cells was calculated and presented here. ACE2 protein levels significantly decreased upon castration in male lungs and increased with testosterone stimulation in lungs of both castrated male and intact female mice (*SI Appendix*, Fig. S5 *D* and *E*). Together, our data indicate that pulmonary *Tmprss2* and *Ace2* expression in bronchial and nonbronchial cells are positively regulated by androgen.

Additionally, we performed costaining experiments with *Ar* and the SARS-CoV-2 entry factors. ISH analysis showed that *Ar* and *Tmprss2* were coexpressed at the cellular level in bronchial cells, and *Ace2* and *Tmprss2* were also coexpressed in bronchial cells (Fig. 2 *G* and *H*). In nonbronchial cells, including alveolar cells, *Tmprss2* and *Ar* were also coexpressed (*SI Appendix*, Fig. S5*F*). *Tmprss2* is partially coexpressed with lung epithelial marker *Sftpb* in both bronchial and alveolar cells (*SI Appendix*, Fig. S5 *G* and *H*). This evidence indicates that SARS-CoV-2 entry factors TMPRSS2 and ACE2 are coexpressed with AR in subsets of pulmonary epithelia, including bronchial and alveolar cells.

### Expression Analysis of AR, *TMPRSS2*, and ACE2 in Human Lungs Shows Higher AR Levels in Males than Females.

**Fig. 3.**
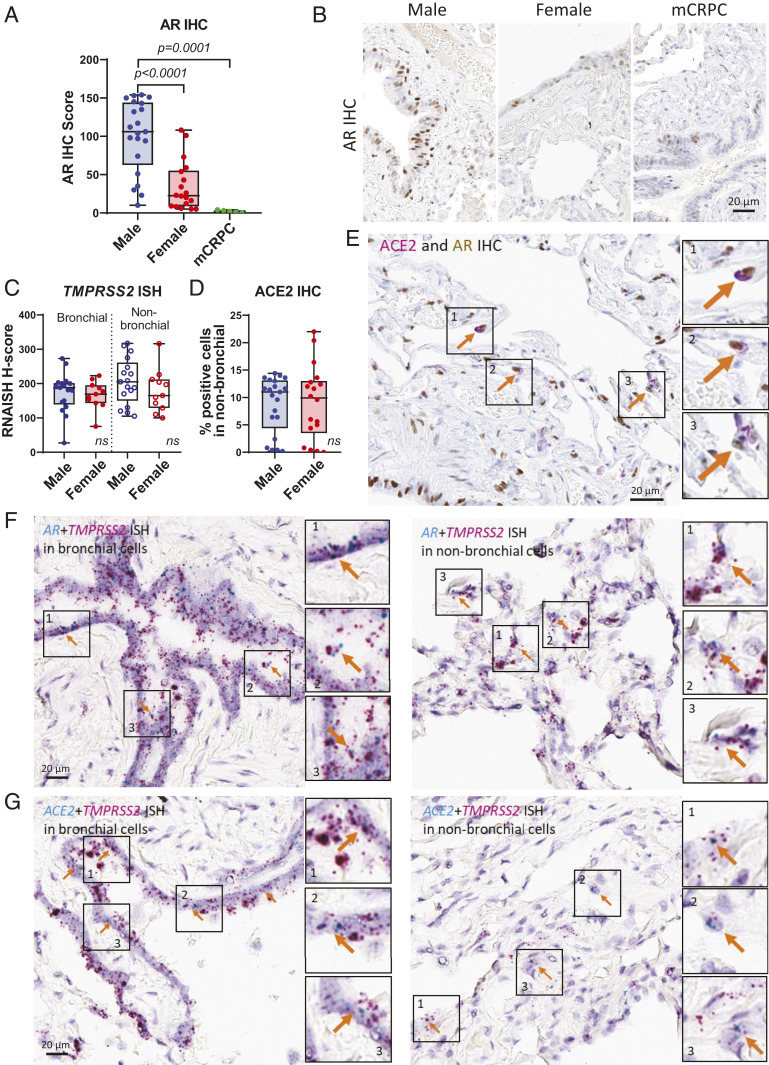
Expression patterns of AR, *TMPRSS2*, and ACE2 in human lungs. (*A*) Quantitative analysis of AR IHC score of human lungs in male (*n* = 21), female (*n* = 18), and mCRPC (*n* = 5). *P* values were calculated by unpaired *t* test. (*B*) Representative images of *A*. (*C*) Quantitative analysis of *TMPRSS2* ISH in bronchial and nonbronchial cells and (*D*) ACE2 IHC in nonbronchial cells of male (*n* = 17) and female (*n* = 11). *P* values were calculated by unpaired *t* test and showed no significance (ns). (*E*) Representative IHC images showing coexpression of ACE2 and AR in human lungs. Arrows indicate cells expressing both ACE2 and AR. (*F*) Representative ISH images showing coexpression of *AR* and *TMPRSS2* and (*G*) *ACE2* and *TMPRSS2* in human bronchial and nonbronchial cells. Arrows indicate cells expressing both (*F*) *AR* and *TMPRSS2* and (*G*) *ACE2* and *TMPRSS2*.

We extended the in situ analysis to human lung tissues using normal lung sections from male, female, and metastatic castration-resistant prostate cancer (mCRPC) patients. AR protein had clear nuclear localization in bronchial and alveolar cells of human lungs. The intensity and quantity of AR protein, represented by AR IHC score, in lungs indicated that males had significantly higher levels of AR than females, whereas mCRPC (of which patients are typically on therapies that systemically suppress testosterone levels) had the lowest AR levels (Fig. 3 *A* and *B*). RNA expression of *TMPRSS2* was examined by ISH on the same cohort and showed bronchial and nonbronchial expression of *TMPRSS2* in human lung tissues (*SI Appendix*, Fig. S6*A*). However, quantitative analysis of *TMPRSS2* ISH did not show significant differences between males and females in either bronchial or nonbronchial cells (Fig. 3*C*). Additionally, ACE2 protein was expressed in nonbronchial cells of human lungs (*SI Appendix*, Fig. S6*B*), but there was no differential expression in males and females (Fig. 3*D*). These results are similar to those observed above in our murine models. Although manipulation of androgen levels with either castration in male mice or supplemented testosterone in both male and female mice led to significant alterations in *Tmprss2* and *Ace2* (and ACE2 protein) levels, intact male and female mice had similar expression levels of these factors (Fig. 2 *D* and *F* and *SI Appendix*, Fig. S5 *C* and *E*).

Dual staining experiments showed that, in alveolar cells, AR and ACE2 protein were coexpressed (Fig. 3*E*) in individual cells. *AR* and *TMPRSS2* RNA were also coexpressed in both bronchial and nonbronchial cells (Fig. 3*F*) shown by dual RNA ISH staining. *ACE2* and *TMPRSS2* RNA were further coexpressed in bronchial and nonbronchial cells (Fig. 3*G*). These data suggest that bronchial and alveolar cells express AR, TMPRSS2, and ACE2 simultaneously; thus, the cellular regulation of *TMPRSS2* and *ACE2* by AR becomes feasible in human lungs.

### Smoking Elevates AR and ACE2 Expressions in Aged Male Lungs.

**Fig. 4.**
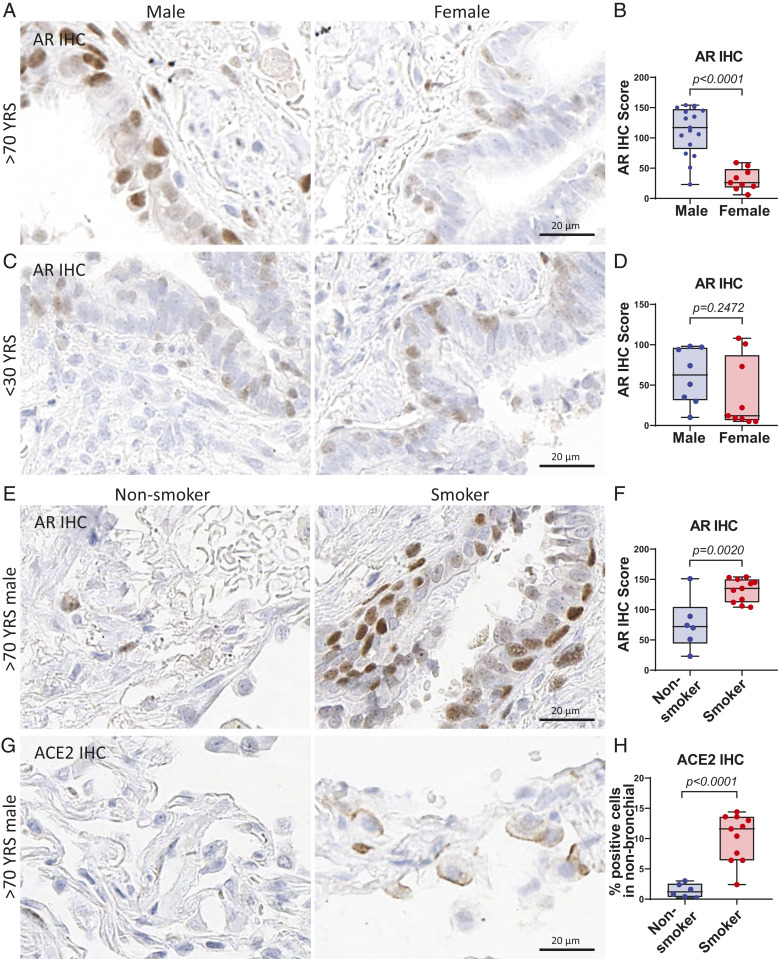
Smoking elevates AR and ACE2 expression in aged male lungs. (*A*) Representative images and (*B*) quantitative analysis of AR IHC score of human lungs in males (*n* = 17) and females (*n* = 9) over 70 y of age. *P* values were calculated by unpaired *t* test in all panels. (*C*) Representative images and (*D*) quantitative analysis of AR IHC score of human lungs in males (*n* = 8) and females (*n* = 9) under 30 y old. (*E*) Representative images and (*F*) quantitative analysis of AR IHC score of aged male (>70 y old) lungs in nonsmokers (*n* = 6) and smokers (*n* = 11). (*G*) Representative images and (*H*) quantitative analysis of ACE2 IHC of aged male (>70 y old) lungs in nonsmokers (*n* = 6) and smokers (*n* = 11).

SARS-CoV-2 has been reported as having higher detrimental impacts in elderly populations, especially men with a smoking history (10). Here, we evaluated the expression of AR and ACE2 in lung tissues from a group of younger (<30 y old) and older (>70 y old) males and females. AR protein had significantly higher expression in males than females in the age group over 70 y old (Fig. 4 *A* and *B* and *SI Appendix*, Fig. S7*A*), whereas similar (relatively lower) expression levels were observed in the under 30-y-old group (Fig. 4 *C* and *D* and *SI Appendix*, Fig. S7*B*). Smoking is a known risk factor for lung disease and a more severe COVID-19 disease course (10); therefore, we examined whether smoking status could alter the expression level of AR and ACE2 in male and female lungs. In an analysis with all age and genders combined, both AR and ACE2 protein had slightly higher expression in smokers than nonsmokers (*SI Appendix*, Fig. S7 *C*–*F*). Importantly, elderly male smokers had significantly higher AR and ACE2 protein expression than nonsmokers in lungs of males over 70 y of age (Fig. 4 *E*–*H* and *SI Appendix*, Fig. S7 *G* and *H*). These data demonstrate that aged males have increased AR and SARS-CoV-2 host entry factor ACE2 protein expression in lungs, and their expression is enhanced by smoking. This suggests that older smoking men have a higher vulnerability to SARS-CoV-2 infection due to elevated entry factor levels.

### AR and BET Antagonists Diminish SARS-CoV-2 Infection and Replication.

**Fig. 5.**
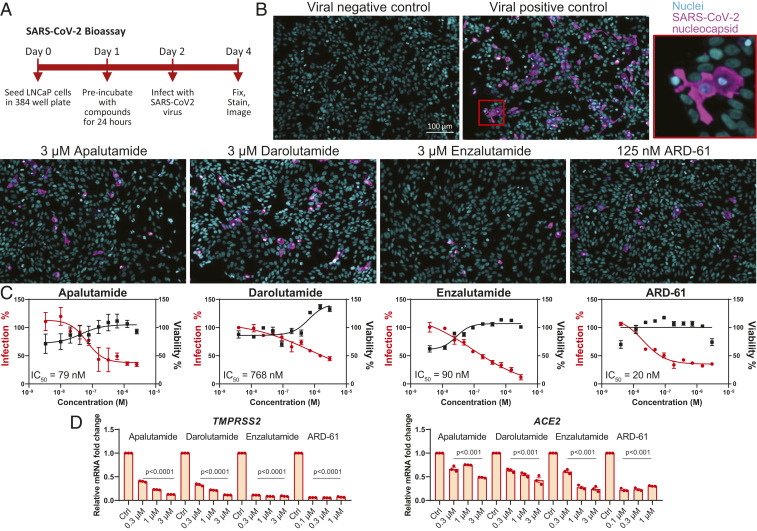
AR antagonists diminish SARS-CoV-2 infection in LNCaP cells. (*A*) Schematic representation of the SARS-CoV-2 bioassay design for the antiviral drug screen. (*B*) Representative images of immunofluorescent staining of nuclei and SARS-CoV-2 nucleocapsid protein in various treatment groups of AR-targeting drugs. (*C*) Dose–response curves of SARS-CoV-2 virus percent infection and cell viability of various AR antagonists in LNCaP. (*D*) Relative levels of *TMPRSS2* and *ACE2* in LNCaP with indicated treatment for 72 h.

In the prostate lineage, our previous chromatin immunoprecipitation sequencing data in LNCaP demonstrated that *TMPRSS2* is a bona fide AR-regulated gene through direct binding of AR, FOXA1, and BRD4 (a BET protein) at the promoter region of *TMPRSS2* (17, 27). Using the same dataset, we found that the *ACE2* promoter also has AR, FOXA1, and BRD4 binding sites, which overlap with open chromatin regions identified by assay for transposase-accessible chromatin using sequencing (*SI Appendix*, Fig. S8), suggesting that *ACE2* is a direct target of AR and BRD4 in the prostate. Here, our data suggest that pulmonary *TMPRSS2* and *ACE2* expression is also mediated by AR signaling in murine and human lungs. Thus, we examined whether blocking AR signaling could impair the infection ability of SARS-CoV-2 in the AR-positive LNCaP prostate cancer cell line, which we found to be permissive to infection with isolated SARS-CoV-2 virus. Using LNCaP cells, we screened Food and Drug Administration (FDA)-approved and experimental small-molecule AR and BET inhibitors for their effects on SARS-CoV-2 infection (Fig. 5*A*). SARS-CoV-2 virus was detected and quantified by high-content immunofluorescence imaging of its nucleocapsid protein, whereas nuclei were counterstained with Hoechst-33342 (Fig. 5*B*). Using this SARS-CoV-2 bioassay platform, we demonstrated that AR antagonists FDA-approved for prostate cancer treatment (apalutamide, darolutamide, enzalutamide) inhibited SARS-CoV-2 infection in LNCaP cells in a dose-dependent manner, with concentration that inhibits response by 50% (IC_50_) values of 79, 768, and 90 nM, respectively. Our experimental small-molecule AR protein degrader ARD-61 (28) also exhibited a dose-dependent reduction in SARS-CoV-2 infectivity, with an IC_50_ of 20 nM (Fig. 5*C*), further demonstrating that AR is indispensable for SARS-CoV-2 infection. In concordance with our data above, *TMPRSS2* and *ACE2* were down-regulated in a dose-dependent manner by AR inhibitors (Fig. 5*D*).

**Fig. 6.**
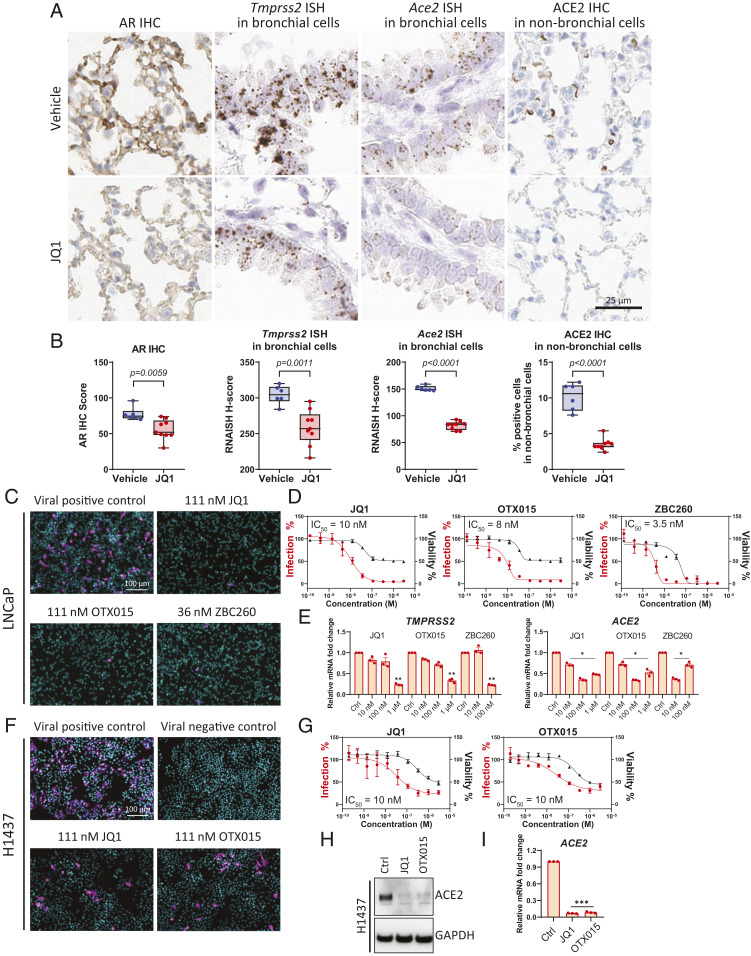
BET antagonists down-regulate AR, ACE2, and *Tmprss2* in murine lung and decrease SARS-CoV-2 infection in human cells. (*A*) Representative images of AR IHC, *Tmprss2* and *Ace2* ISH in bronchial cells, and ACE2 IHC in nonbronchial cells in murine lungs, from vehicle and JQ1-treated male mice. (*B*) Quantitative analysis of AR IHC score, *Tmprss2* and *Ace2* ISH H score, and percentage of ACE2 positive cells in 500 nonbronchial cells of murine lung from vehicle (*n* = 6) and JQ1 (*n* = 9) treated male mice. *P* values were calculated by unpaired *t* test. (*C*) Representative images of immunofluorescent staining of nuclei and SARS-CoV-2 nucleocapsid protein in various treatment groups of BET inhibitors in the SARS-CoV-2 bioassay in LNCaP cells. (*D*) Dose–response curves of SARS-CoV-2 virus percent infection and cell viability of LNCaP cells treated with various BET antagonists. (*E*) Relative levels of *TMPRSS2* and *ACE2* in LNCaP cells with indicated treatment for 72 h. *P* values were calculated by unpaired *t* test. *: *P* < 0.05; **: *P* < 0.01. (*F*) As in *C*, but in H1437 cells. (*G*) As in *D*, but in H1437 cells. (*H*) Protein levels of ACE2 with control (Ctrl), 1 µM JQ1, or 1 µM OTX015 treatment for 72 h in H1437 cells. (*I*) Relative levels of *ACE2* in H1437 cells with indicated treatment for 72 h. JQ1 (1 µM), OTX015 (1 µM). ***: *P* < 0.001. *P* values were calculated by unpaired *t* test.

Furthermore, we previously reported that androgen signaling in prostate cancer requires BET protein activity (17) and identified a BRD4 binding site in the *TMPRSS2* and *ACE2* genes (*SI Appendix*, Fig. S8); thus, we examined whether pulmonary AR, *Tmprss2*, and *Ace2* levels were responsive to systemic treatment with BET inhibitor JQ1 in male mice. After 5 d, individual lungs were collected from intact C57BL/6 male mice treated with either vehicle or 50 mg/kg JQ1 (*SI Appendix*, Fig. S9*A*). Levels of *Tmprss2* and *Ace2* were then examined using bulk lung tissue harvested from individual mice. The results showed significant transcriptional repression of *Tmprss2* and *Ace2* by JQ1 in this bulk lung tissue analysis (*SI Appendix*, Fig. S9*B*), which we did not observe using bulk lung tissue in intact and castrated male mice (*SI Appendix*, Fig. S4*C*). RNA ISH of the same cohort confirmed that *Tmprss2* and *Ace2* levels were significantly lower in mice treated with JQ1 compared to vehicle in bronchial cells (Fig. 6 *A* and *B* and *SI Appendix*, Fig. S9*C*). Furthermore, ACE2 IHC in lungs demonstrated that ACE2 protein levels were reduced by JQ1 in nonbronchial cells (Fig. 6 *A* and *B* and *SI Appendix*, Fig. S9*D*). Protein levels of AR measured by AR IHC score were lower in lungs of JQ1-treated mice (Fig. 6 *A* and *B* and *SI Appendix*, Fig. S9*D*).

Together, these data suggested that, like AR-targeting compounds, BET inhibitors may possess therapeutic potential for mitigating SARS-CoV-2 infection. We thus used the SARS-CoV-2 bioassay platform to evaluate the antiviral effects of BET inhibitors (JQ1, OTX015) and a BET protein degrader (ZBC260) (29). JQ1, OTX015, and ZBC260 all decreased the infectivity of SARS-CoV-2 in LNCaP cells, with IC_50_ values of 10, 8, and 3.5 nM, respectively (Fig. 6 *C* and *D*), and BET inhibitor or degrader treatment led to associated decreases in *TMPRSS2* and *ACE2* (Fig. 6*E*). Similarly, in H1437 cells originating from the lung lineage, JQ1 and OTX015 decreased the infectivity of SARS-CoV-2, with IC_50_ values of 10 nM for both compounds (Fig. 6 *F* and *G*), and resulted in associated decreases in ACE2 protein and mRNA levels (Fig. 6 *H* and *I*). These data suggest that BET proteins may play a role in regulating SARS-CoV-2 entry factor *ACE2* expression independent of AR regulation, thus supporting the notion that BET inhibition may have a broad therapeutic range in mitigating SARS-CoV-2 infection.

## Discussion

ACE2 and TMPRSS2 were shown to be the key mediators of SARS-CoV-2 viral entry early in the COVID-19 pandemic, but which cells of the upper airway tract express them and mechanisms governing their expression in the lung were not defined (3, 4). Here, through snRNAseq, ISH, and IHC methodologies, TMPRSS2 and ACE2 expression was identified in specific alveolar and bronchial epithelial cell types, as well as coexpression with AR. Similar to the prostate, *TMPRSS2* expression was found to be regulated by androgens in certain cells in the lungs, and we also identified *ACE2* as an AR-regulated target; these findings support an early hypothesis that differences in androgen levels may be one reason for gender disparities in COVID-19 outcomes (12). Analysis of other variables here also showed that ACE2 and AR levels were elevated in older male smokers, providing a possible link between smoking and age risk factors with a more severe COVID-19 disease course (10).

**Fig. 7.**
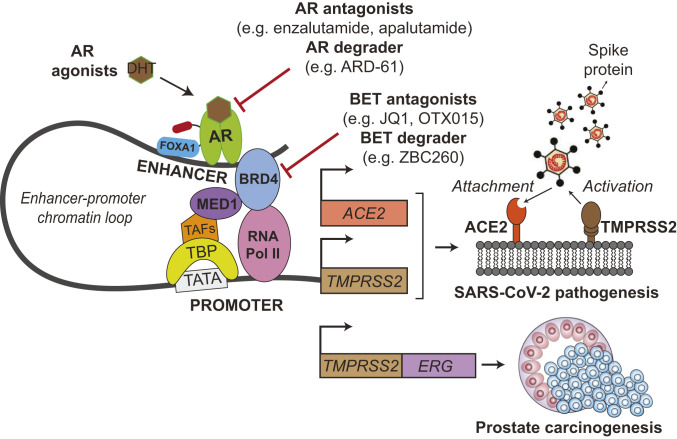
Schematic illustration of *TMPRSS2* and *ACE2* regulation by AR and their roles during SARS-CoV-2 infection. AR binds to enhancer elements of both *ACE2* and *TMPRSS2* genes, connecting the regulatory circuit between the enhanceosome complex (comprising MED1, BRD4, etc.) and the promoter-bound RNA polymerase machinery (comprising TBP, TAFs, etc.) to activate gene expression. AR regulation of the driver *TMPRSS2-ERG* oncogenic gene fusion has been thoroughly credentialed in prostate cancer. During SARS-CoV-2 infection, the serine protease TMPRSS2 primes the viral Spike protein, which then binds to the ACE2 receptor to gain entry into host cells. We demonstrate that AR regulates *TMPRSS2* and *ACE2* expression in prostate and subsets of pulmonary epithelial cells. Agents directly targeting AR or BET proteins inhibit SARS-CoV-2 infectivity through transcriptional down-regulation of host cell *TMPRSS2* and *ACE2* expression. TBP, TATA-binding protein; TAFs, TBP-associated factors; FOXA1, forkhead box A1; BRD4, bromodomain-containing protein 4; MED1, mediator complex subunit 1.

Importantly, we find that targeting the transcriptional regulation of *ACE2* and *TMPRSS2* may be an attractive treatment approach for COVID-19 (Fig. 7). Our data show that strategies that decrease AR signaling, either through AR antagonists already FDA-approved for prostate cancer treatment [enzalutamide, apalutamide, darolutamide (30)], AR degraders [ARD-61 (28)], or castration, can lead to dose-dependent decreases in *TMPRSS2* and *ACE2* expression and attenuate SARS-CoV-2 infectivity. As an important caveat, we relied upon LNCaP prostate cancer cells for the SARS-CoV-2 bioassay with AR inhibitors, since we were unable to identify a lung epithelial cell line susceptible to SARS-CoV-2 infection that also had adequate AR signaling. LNCaP cells are dependent on AR and express the protein at high levels, so it is possible that AR signaling and subsequent targeting efficacy may be different in the lung epithelial cells of COVID-19 patients.

Likewise, BET protein small-molecule inhibitors [JQ1, OTX015—which has been studied clinically in the context of cancers (31)] or degraders [ZBC260 (29)] also decrease expression of viral host entry factors and prevent SARS-CoV-2 infection. This could result from the known coactivator role that BET proteins play in mediating AR activity (17) or through the AR-independent roles of BET proteins in transcriptional regulation (31). It is also important to note that, while we demonstrate androgen regulation of ACE2, TMPRSS2, and AR in subsets of lung epithelial cells, our data do not suggest that all cells in the lung control these factors through AR signaling. For instance, JQ1 and OTX015 decrease SARS-CoV-2 infectivity and ACE2 levels in H1437 lung adenocarcinoma cells (Fig. 6 *F*–*I*), but androgens and AR antagonists did not decrease *ACE2* in this cell line (*SI Appendix*, Fig. S1*D*). Thus, in cell types where AR regulation of *ACE2* and *TMPRSS2* does not exist, the broader transcriptional repression by BET inhibitors may block expression of these key host factors, and agents targeting BET proteins may have a different therapeutic efficacy in COVID-19 than direct AR inhibitors. BET inhibitors can also affect innate and adaptive immune responses, through processes such as repression of *IFNG* (IFN gamma) (32), which could potentially attenuate the cytokine storm associated with COVID-19.

Several clinical trials are already underway to evaluate targeting of androgen signaling for treatment of COVID-19 (10). Some of these studies are examining the efficacy of the AR antagonists enzalutamide (NCT04456049, NCT04475601) or bicalutamide (NCT04509999, NCT04374279) in COVID-19 patients. The Hormonal Intervention for the Treatment in Veterans with COVID-19 Requiring Hospitalization trial in male veterans with COVID-19 is employing degarelix, a gonadotropin-releasing hormone antagonist that rapidly suppresses serum testosterone levels (NCT04397718). The 5-alpha reductase inhibitor dutasteride, which converts testosterone into the more potent AR ligand dihydrotestosterone, is further under investigation (NCT04446429). No clinical trials of BET inhibitors in COVID-19 have been initiated at the time of this publication, but several compounds have been advanced to the clinic for other indications such as cancer, including OTX015/MK-8628 highlighted in our study, and possess the potential to be quickly repurposed for COVID-19 (31). Although not formally initiated, Resverlogix has announced plans for a COVID-19 phase 2 clinical trial with their BET inhibitor, apabetalone, which was recently tested in a phase 3 study for cardiovascular disease (33).

A plethora of clinical trials are also underway to examine compounds that inhibit the function of TMPRSS2 or ACE2 (10, 34). For example, numerous studies are evaluating the utility of protease inhibitors of TMPRSS2, alone or in combination with other agents, in COVID-19 patients; these include camostat (11 clinical trials initiated at time of publication, such as NCT04353284, NCT04524663, NCT04470544), nafamostat (NCT04352400, NCT04418128, NCT04390594, NCT04473053), and bromhexine (NCT04424134, NCT04355026, NCT04273763, NCT04340349, NCT04405999). Notably, TMPRSS2 has also been shown to cleave the hemagglutinin protein of influenza viruses, a step necessary for influenza infectivity. Similar to coronavirus models, *TMPRSS2* knockout mice exhibit reduced lung pathogenesis and decreased viral spread after infection with different strains of influenza virus, including H1N1, the virus responsible for the 2009 swine flu pandemic (35, 36). Therapeutics targeting TMPRSS2 expression or activity may, therefore, be beneficial not only for coronavirus-infected patients but also those infected with influenza.

Although our findings show a significant role of androgens in mediating expression of *TMPRSS2* and *ACE2*, the full array of mechanisms responsible for the gender disparities observed in COVID-19 outcomes is likely multifactorial. For instance, males and females have differences in innate immune responses and susceptibility to viral infections in general (10, 11). Estrogen signaling, via the estrogen receptor, controls a complex network of immune response genes, and it has been postulated that estrogens play a protective role in preventing the cytokine storm that can occur in the later stages of COVID-19 (37). Androgens also control genes involved in the immune response but generally support a more immunosuppressive state (38). Our study also finds that AR levels are higher in older males (greater than 70 y of age) compared to females, and smoking increases AR and ACE2 expression in males over 70 y old. Behavioral factors, such as smoking, and comorbid conditions that are more prevalent in males (e.g., hypertension, chronic obstructive pulmonary disease, diabetes) may be contributing factors to the increased mortality and hospitalization rates observed in men through mechanisms in addition to those highlighted in our study (10, 11, 39, 40). Finally, apart from bronchial epithelium and lung which were investigated in our cohort, cells lining the nasopharyngeal airway also might play a role in virus infection and sustenance.

*TMPRSS2-ERG* has long been studied by the prostate cancer field as an oncogenic gene fusion under the control of the androgen-regulated *TMPRSS2* promoter expressed in the majority of prostate cancers (Fig. 7). Due to the COVID-19 pandemic, TMPRSS2 has gained notability in a different realm as the priming factor for SARS-CoV-2 Spike protein following attachment to ACE2. Our studies presented herein provide a strong rationale for the use of AR or BET inhibitors in COVID-19 treatment to decrease *TMPRSS2* and *ACE2* expression, and the results of the many clinical studies mentioned above are eagerly awaited.

## Methods

### Cell Culture.

H1437, HCC4006, Caco-2, Calu-3, and LNCaP cells were obtained from American Type Culture Collection (ATCC) and maintained under 5% CO_2_ at 37 °C in medium according to ATCC. HPAEpiC (human pulmonary alveolar epithelial cells) were purchased from ScienCell Research Laboratories. All cell lines were tested negative for mycoplasma and authenticated by genotyping.

### Compounds.

Apalutamide, darolutamide, dexamethasone, enzalutamide, mifepristone, OTX015, and JQ1 were purchased from Selleckchem. R1881, dihydrotestosterone (DHT), and beta-estradiol were purchased from Sigma-Aldrich. BET protein degrader ZBC260 and AR protein degrader ARD-61 were described previously (28, 29).

### IHC.

IHC was performed on 4-μm-thick formalin-fixed, paraffin-embedded (FFPE) tissue sections using anti-AR rabbit monoclonal primary antibody (prediluted, pH 9, catalog no. 760-4605, Roche-Ventana) and anti-ACE2 rabbit monoclonal primary antibody (1:100, pH 9, catalog no. GTX01160, GeneTex). IHC was carried out on the Benchmark XT automated slide staining system (Roche-Ventana Medical Systems) using the UltraView Universal diaminobenzidine (DAB) detection kit (catalog no. 760-500, Roche-Ventana) and Hematoxylin II (catalog no. 790-2208, Roche-Ventana) for counterstain. Dual IHC was performed consecutively, and signals were developed using the Universal DAB detection kit and the Discovery purple kit (catalog no. 760-229, Roche-Ventana). Staining was evaluated under 100× and 200× magnification using a bright-field microscope. See *SI Appendix* for scoring details.

### RNA ISH.

RNA ISH was performed on 4-μm-thick FFPE tissue sections using RNAscope 2.5 high definition (HD) Brown kit (Advanced Cell Diagnostics) for single target and RNAscope 2.5 HD Duplex kit (322430) for dual targets. RNA quality was evaluated using a positive control probe against human *PPIB*. Assay background was monitored using a negative control probe against bacterial gene DapB. RNA ISH was performed as previously described (41, 42). FFPE tissue sections were baked, deparaffinized in xylene, and dehydrated in 100% ethanol. After hydrogen peroxide pretreatment and heat-induced target retrieval, tissue samples were permeabilized using protease and hybridized with target probe followed by a series of signal amplification steps. Chromogenic detection was performed using DAB for single target or a consecutive combination of horseradish peroxidase-based Green and alkaline phosphatase-based Fast Red chromogens, followed by 50% Gill’s Hematoxylin I (Fisher Scientific) counterstain. Staining was evaluated under 100× and 200× magnification using a bright-field microscope. See *SI Appendix* for probes and scoring information.

### SARS-CoV-2 Infection Bioassay.

LNCaP and H1437 cells were grown in Roswell Park Memorial Institute (RPMI) 1640 supplemented with 10% fetal bovine serum and seeded at 8,000 cells per well in poly-D-lysine−coated 384-well plates (Perkin-Elmer, 6057300), or 10,000 cells per well in 96-well plates (Corning, 3603), respectively. Cells were allowed to attach and recover for 12 h to 18 h. BET and AR antagonists were solubilized in dimethyl sulfoxide (DMSO) and dispensed (0.1 nM to 10 µM, 10-point dilution series, *n* = 3) using an HPD300e digital compound dispenser and allowed to incubate 24 h prior to infection, with a final DMSO vehicle concentration of 0.1%. SARS-CoV-2 WA1/2020 strain (BEI resources catalog no. NR-52281) was added in BSL3 containment at a final working dilution equivalent to a multiplicity of infection of 10 and was allowed to incubate for 48 h at 37 °C. Wells were fixed with 4% paraformaldehyde, permeabilized with 0.03% Triton X-100, and blocked with antibody buffer (1.5% bovine serum albumin [BSA], 1% goat serum, 0.0025% Tween-20). Following blocking, plates were sealed, surface decontaminated, and transferred to a BSL2 laboratory for staining. Cells were stained overnight with SARS-CoV-2 nucleoprotein primary antibody (ProSci catalog no. 35-579, 1:2,000) and then stained with anti-mouse IgG:AlexaFluor 647 secondary (Invitrogen catalog no. A21235, 1:1,000) and Hoechst 33342 (Invitrogen catalog no. H3570, 1:2,000).

### High-Content Imaging and Analysis of SARS-CoV-2−Infected Cells.

Plates were imaged on a Thermo-Fisher CX5 high-content microscope with a UPlanFLN 10×/0.3NA objective. Nine fields were acquired per well for each of the two fluorescent channels (Hoechst-386/23 nm, N-protein-650/13 nm). Images were analyzed using CellProfiler to quantify the percentage of infected cells at the well level (43). Infected cell areas were first identified by two-class Otsu segmentation in the N-protein image. Nuclei were then identified in a similar manner and were related to infected cell areas using the relate objects module. Infected cells were identified if a nucleus was residing within an infected cell area, and percentages per well were calculated from the infected cell/total cell count. Dose–response curves were fit and IC_50_ values tabulated using the four-parameter logistic model in Graphpad Prism. Plate-based normalization was performed using 32 infected (0% effect) and 32 uninfected (100% effect) control wells (mean LNCaP infectivity range: 18 to 25%). Viability was assessed by comparing cell counts in treated wells to the average cell count in the 32 uninfected control wells (100% viability).

### Analysis of Published Human and Mouse Single-Cell Sequencing Datasets.

To evaluate *ACE2*/*TMPRSS2*/*AR* expression in lung tissues at the cell level, we searched for publicly available scRNAseq datasets based on the following criteria: 1) profiled healthy human and/or mouse lung tissues; 2) generated with 10× Chromium platform; and 3) libraries prepared from single cells. Four human datasets and three mouse datasets were selected (19–23, 25, 44–46). Raw unique molecular index (UMI) counts were downloaded from Gene Expression Omnibus (GEO). For datasets that included samples from patients, only cells from healthy controls were extracted. Data processing (including normalization and identification of highly variable genes) and integration were performed with Seurat v3 (46). To standardize cell annotation across datasets, label transferring was applied using annotations from one dataset as reference (Habermann study for human, Raredon study for mouse). Only cells with high-confidence label prediction (max prediction score of ≥0.9) were used to evaluate cell type-specific expression of *ACE2*/*TMPRSS2*/*AR*. Log-normalized expression value by Seurat was used to generate the bubble plot. For the published prostate scRNAseq dataset (25), fastq files from samples collected at day 0 (intact prostate) and 14 d postcastration were downloaded from sequence read archive and processed with Cell Ranger (3.1.0); prebuilt mm10-2.1.0 provided by 10× Genomics was used as the reference genome. Cell annotation was downloaded from https://singlecell.broadinstitute.org/single_cell/study/SCP859. Log-normalized expression value by Seurat v3 was used to generate the violin plots. See *SI Appendix* for data analysis of the lung and prostate comparisons.

### Statistical Analysis.

Sample sizes are listed on the figures or in figure legends. *P* values were calculated by GraphPad Prism 8 using two-tailed unpaired *t* test, and exact *P* values are provided.

## Supplementary Material

Supplementary File

## Data Availability

The snRNAseq data have been deposited in the GEO database, https://www.ncbi.nlm.nih.gov/geo (accession no. GSE159576). All study data are included in the article and *SI Appendix*.
