# Supplementary material for: Targeting transcriptional regulation of SARS-CoV-2 entry factors ACE2 and TMPRSS2
Source: Proc Natl Acad Sci U S A. 2020 Dec 28;118(1):e2021450118. doi: 10.1073/pnas.2021450118 (PMC7817128; doi:10.1073/pnas.2021450118)
Supplement: Supplementary File [file pnas.2021450118.sapp.pdf]

SUPPLEMENTARY INFORMATION

Targeting Transcriptional Regulation of SARS-CoV-2 Entry Factors *ACE2* and *TMPRSS2*

Yuanyuan Qiao[1,2,7*], Xiao-Ming Wang[1,2,*], Rahul Mannan[1,2,*], Sethuramasundaram Pitchiaya[1,2], Yuping Zhang[1,2], Jesse W. Wotring[3], Lanbo Xiao[1,2], Dan R. Robinson[1,2], Yi-Mi Wu[1,2], Jean Ching-Yi Tien[1,2], Xuhong Cao[1,2,4], Stephanie A. Simko[1,2], Ingrid J. Apel[1,2], Pushpinder Bawa[1,2], Steven Kregel[1,2], Sathiya P. Narayanan[1], Gregory Raskind[1], Stephanie J. Ellison[1], Abhijit Parolia[1,2], Sylvia Zelenka-Wang[1,2], Lisa McMurry[1,2], Fengyun Su[1], Rui Wang[1], Yunhui Cheng[1], Andrew D. Delekta[1], Zejie Mei[5], Carla D. Pretto[6], Shaomeng Wang[1,3,6,7,8], Rohit Mehra[1,2,7,#], Jonathan Z. Sexton[3,6,9,10,#], and Arul M. Chinnaiyan[1,2,4,7,11,#]

[1] Michigan Center for Translational Pathology, University of Michigan, Ann Arbor, MI, 48109, USA
[2] Department of Pathology, University of Michigan, Ann Arbor, MI, 48109, USA
[3] Department of Medicinal Chemistry, College of Pharmacy, University of Michigan, Ann Arbor, MI, 48109, USA
[4] Howard Hughes Medical Institute, University of Michigan, Ann Arbor, MI, 48109, USA
[5] State Key Laboratory of Cell Biology, CAS Center for Excellence in Molecular Cell Science, University of Chinese Academy of Sciences, Shanghai, 200031, China
[6] Department of Internal Medicine, University of Michigan, Ann Arbor, MI, 48109, USA
[7] Rogel Cancer Center, University of Michigan, Ann Arbor, MI, 48109, USA
[8] Department of Pharmacology, University of Michigan, Ann Arbor, MI, 48109, USA
[9] Center for Drug Repurposing, University of Michigan, Ann Arbor, MI, 48109, USA
[10] Michigan Institute for Clinical and Health Research, University of Michigan, Ann Arbor, MI, 48109, USA
[11] Department of Urology, University of Michigan, Ann Arbor, MI, 48109, USA
* These authors contributed equally
[#] Co-senior authors

Correspondence to:
Arul M. Chinnaiyan, M.D., Ph.D.
arul@umich.edu

This PDF file includes:
Figures S1 to S9
Supplementary methods
Supplementary information references

www.pnas.org/cgi/doi/10.1073/pnas.2021450117

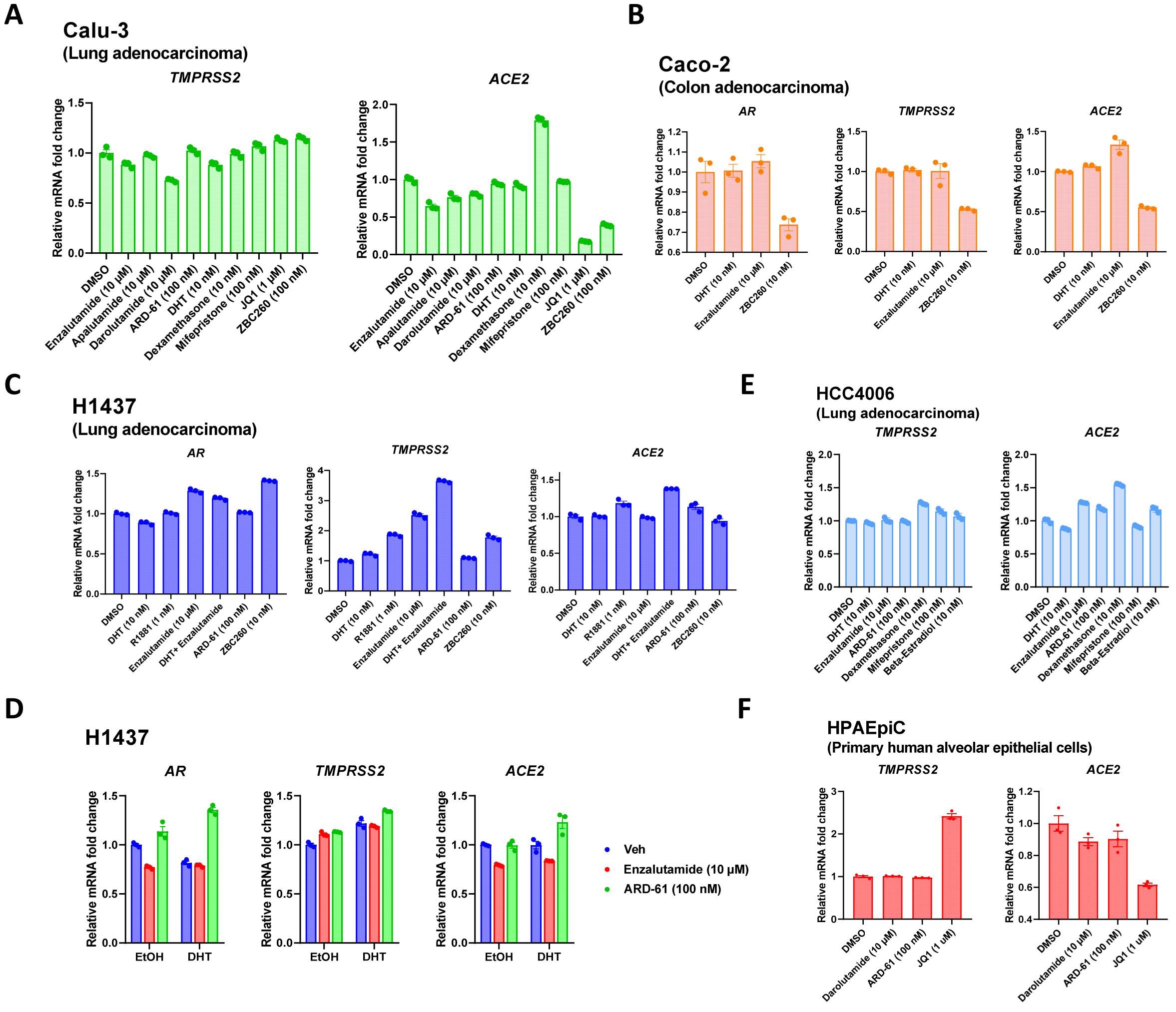

Figure S1. *AR*, *TMPRSS2,* and *ACE2* expression in human cell lines. (A) Relative messenger RNA (mRNA) levels of *TMPRSS2* and *ACE2* in Calu-3 cells with indicated treatment for 48 hours. (B) Relative mRNA levels of *AR*, *TMPRSS2,* and *ACE2* in Caco-2 cells with indicated treatment for 48 hours. (C) Relative mRNA levels of *AR*, *TMPRSS2,* and *ACE2* in H1437 cells with various treatment for 48 hours. (D) Relative mRNA levels of *AR*, *TMPRSS2,* and *ACE2* in H1437 cells with indicated treatment for 48 hours. (E) Relative mRNA levels of *TMPRSS2* and *ACE2* in HCC4006 cells with various treatment for 48 hours. (F) Relative mRNA levels of *TMPRSS2* and *ACE2* in HPAEpiC cells with various treatment for 48 hours.

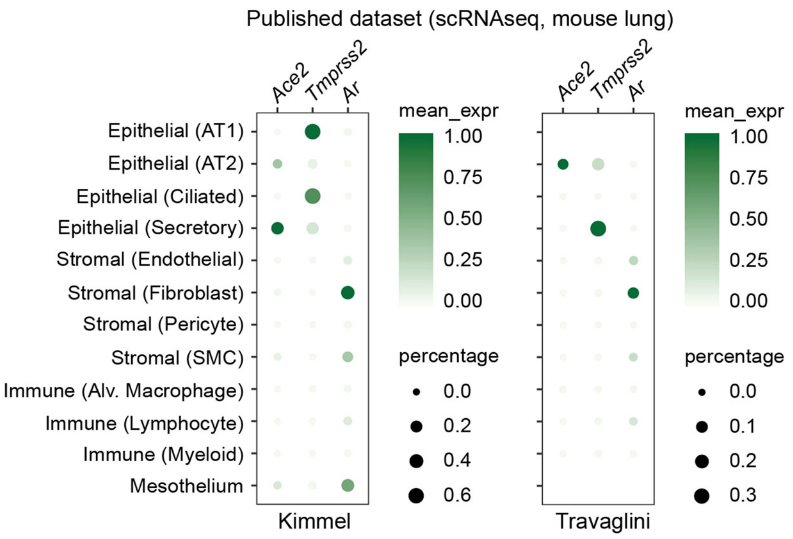

Figure S2. Additional single cell analyses of mouse lung from published datasets. Bubble plot of *Ace2*, *Tmprss2,* and *Ar* expression from two additional publicly available scRNAseq datasets of mouse lung (1, 2). Color bar represents mean expression of each gene in specific cell types, and bubble size represents the percentage of cells in each cell type that expresses the appropriate gene. Plot is labeled below with an identifier, reflecting the name of the first author of the appropriate manuscript.

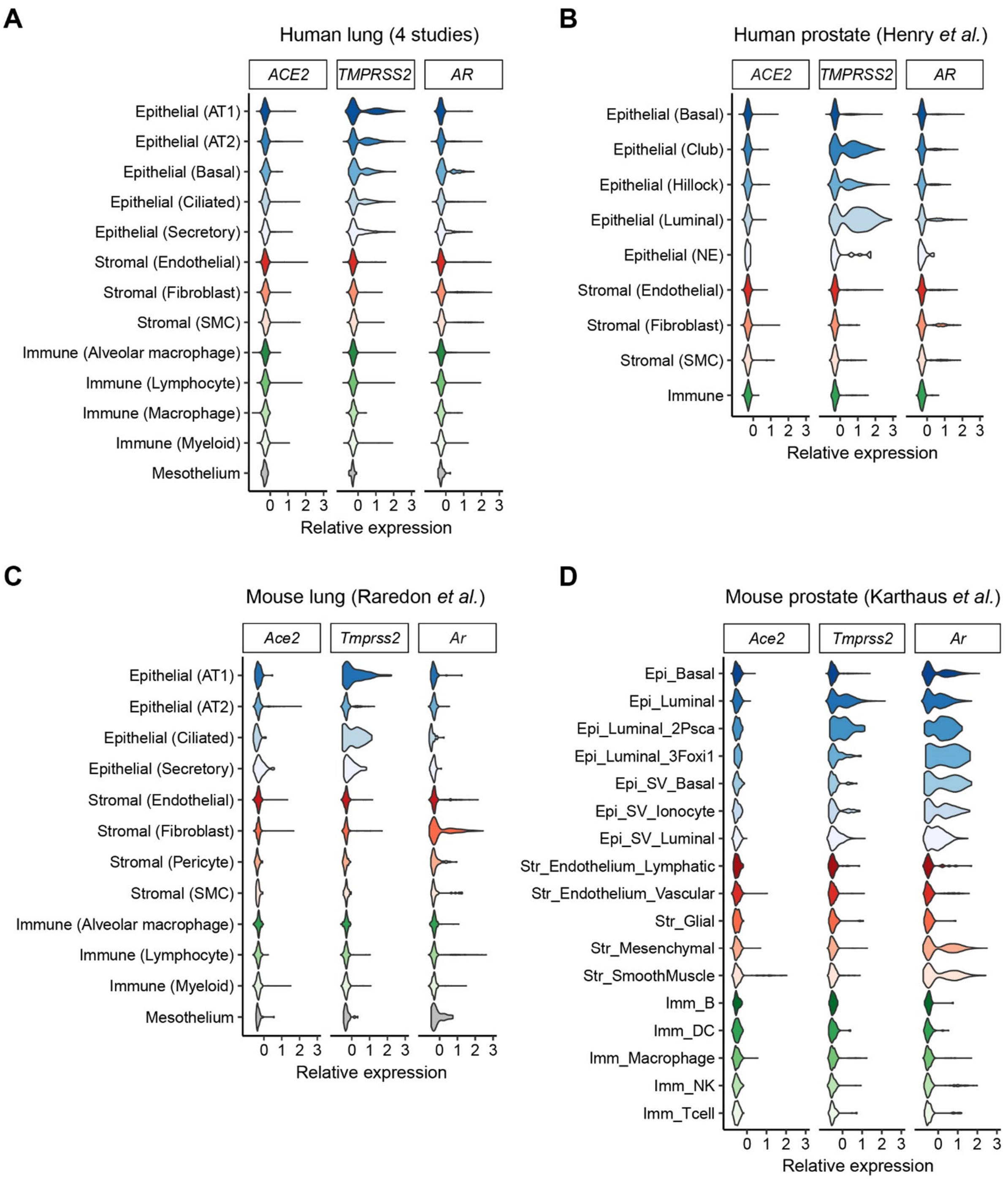

Figure S3. Relative expression levels of *ACE2*, *TMPRSS2*, and *AR* across cell types in lung and prostate. (A) Violin plot representing relative expression of *ACE2*, *TMPRSS2*, and *AR* in the human lung from four publicly available single cell datasets (2-5). (B) Violin plot representing relative expression of *ACE2*, *TMPRSS2*, and *AR* in the human prostate from one public single cell dataset (6). (C)Violin plots representing relative expression of *Ace2*, *Tmprss2*, and *Ar* in mouse lung from a publicly available single cell dataset (4). (D) Violin plots representing relative expression of *Ace2*, *Tmprss2*, and *Ar* in mouse prostate from a publicly available single cell dataset (7).

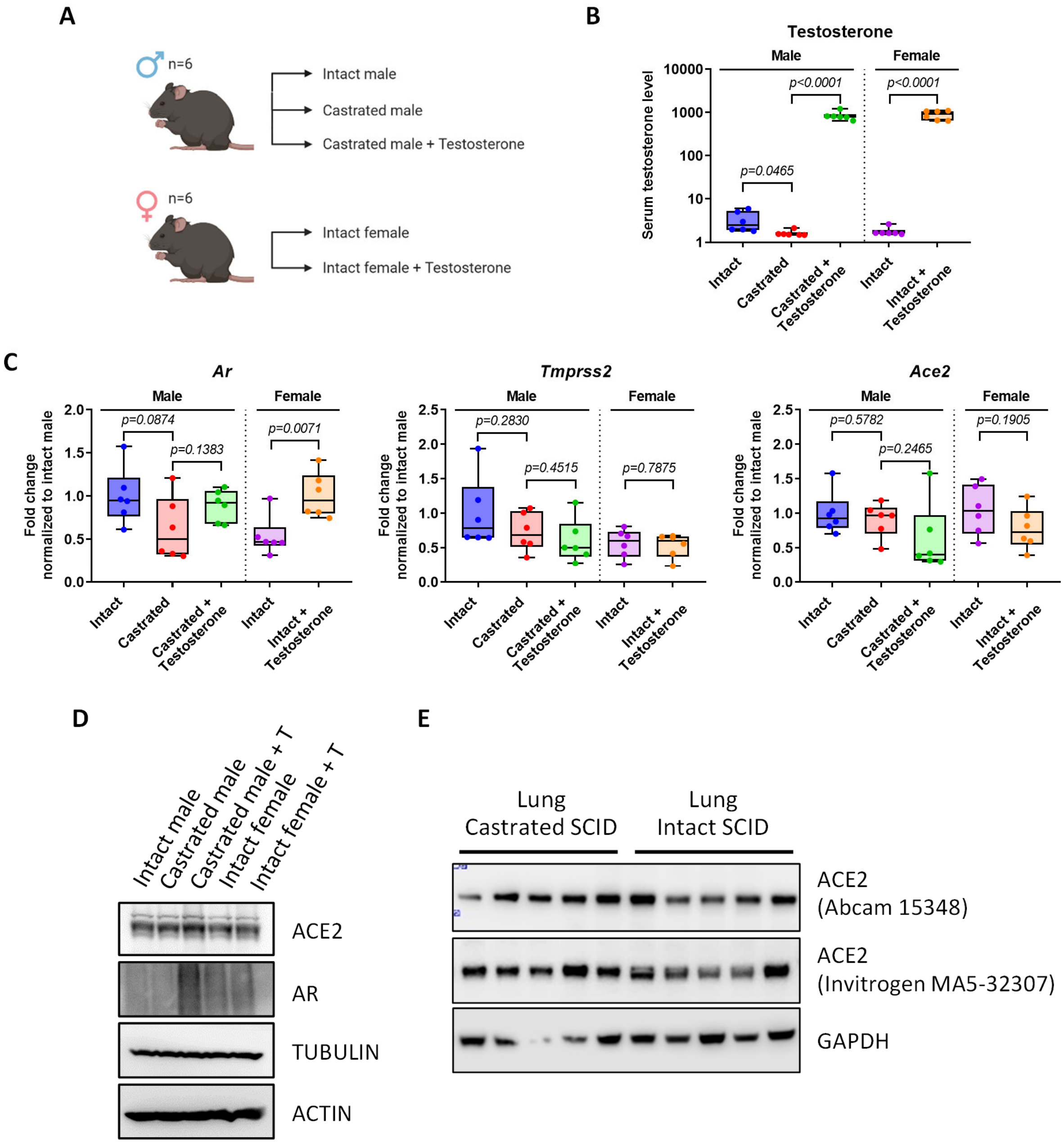

Figure S4. Murine study of male and female mice with various testosterone levels. (A) Murine study design of C57BL/6 male mice for intact, castrated, and castrated + testosterone; female mice for intact, intact + testosterone. (B) Serum testosterone (T) levels in indicated groups. N=6 in each group. *P* values were calculated by unpaired *t* test. (C) Relative mRNA levels of *Ar*, *Tmprss2*, and *Ace2* of murine lungs in indicated groups. N=6 in each group. *P* values were calculated by unpaired *t* test. (D) Protein levels of angiotensin-converting enzyme 2 (ACE2), androgen receptor (AR), and loading controls of murine lungs in indicated groups. (E) Protein levels of ACE2 of individual murine lungs of severe combined immunodeficiency (SCID) mice.

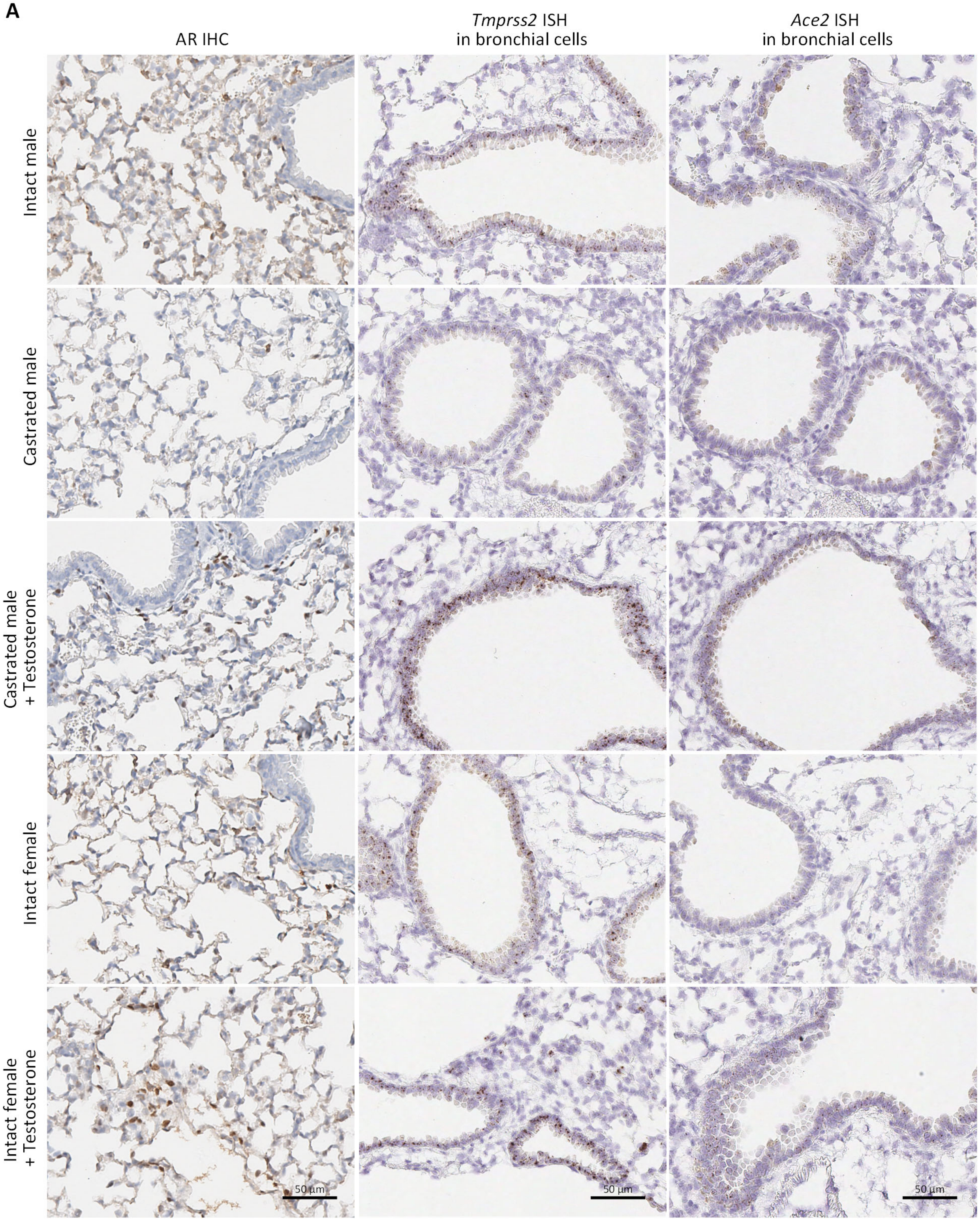
A
AR IHC
Tmprss2 ISH
in bronchial cells
Ace2 ISH
in bronchial cells
Intact male
Castrated male
Castrated male
+ Testosterone
Intact female
Intact female
+ Testosterone
50 µm
50 µm
50 µm

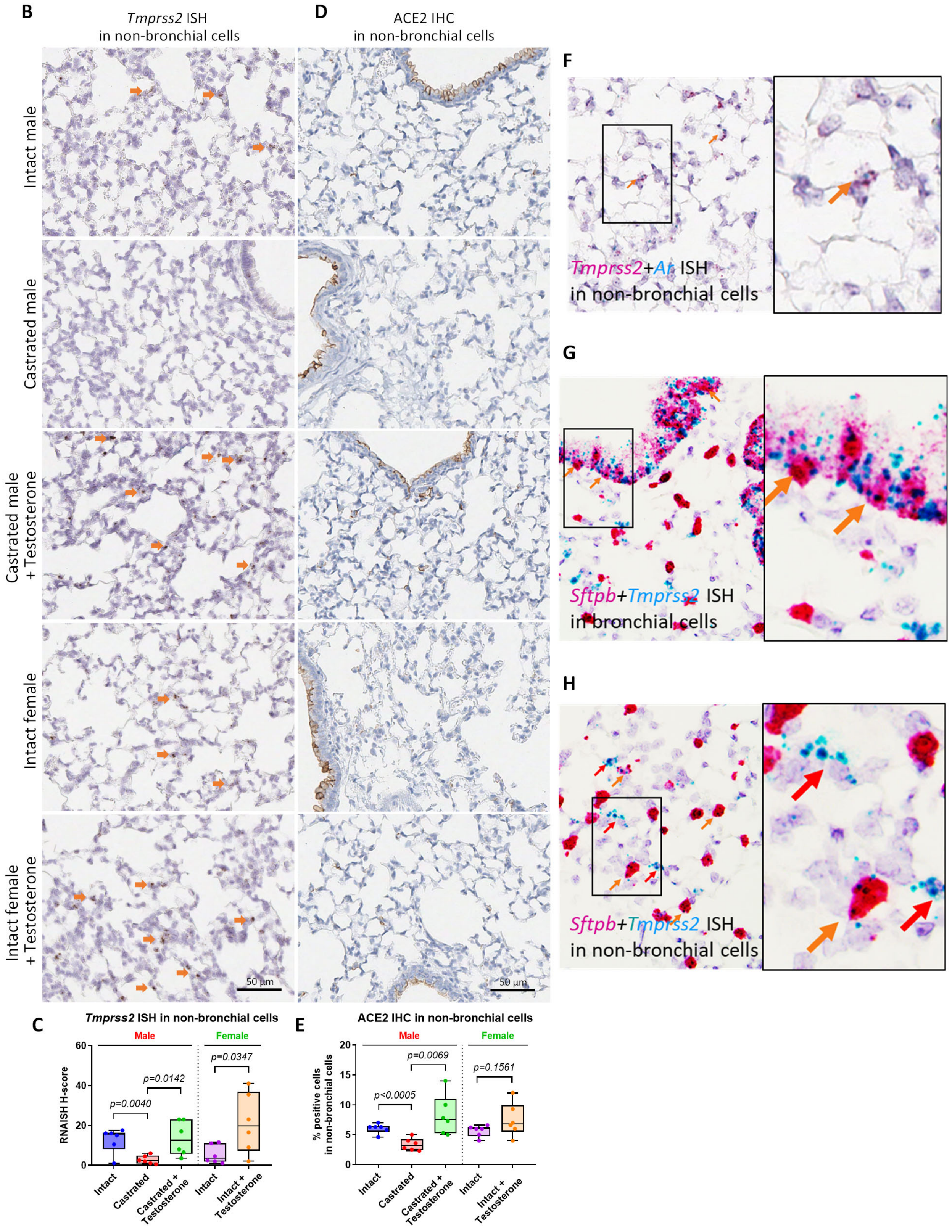
B
Tmprss2 ISH in non-bronchial cells
D
ACE2 IHC in non-bronchial cells
Intact male
Castrated male
Castrated male + Testosterone
Intact female
Intact female + Testosterone
50 µm
F
Tmprss2+Ar ISH in non-bronchial cells
G
Sftpb+Tmprss2 ISH in bronchial cells
H
Sftpb+Tmprss2 ISH in non-bronchial cells
C
Tmprss2 ISH in non-bronchial cells
Male
Female
RNAISH H-score
p=0.0040
p=0.0142
p=0.0347
Intact
Castrated
Castrated + Testosterone
Intact + Testosterone
E
ACE2 IHC in non-bronchial cells
% positive cells in non-bronchial cells
p<0.0005
p=0.0069
p=0.1561

Figure S5. Expression levels of ACE2 and *Tmprss2* in murine lungs are regulated by androgen. (A) Representative images of AR immunohistochemistry (IHC), *Tmprss2* RNA *in situ* hybridization (ISH), and *Ace2* ISH of murine lungs from male (intact, castrated, castrated + testosterone) and female (intact, intact + testosterone). Increased magnification of the same images for intact and castrated male are shown in Figure 2A, C, and E. (B) Representative images of *Tmprss2* ISH in non-bronchial cells of murine lungs from male (intact, castrated, castrated + testosterone) and female (intact, intact + testosterone). (C) Quantitative analysis of *Tmprss2* ISH H-score in non-bronchial cells of murine lungs from indicated groups. *P* values were calculated by unpaired *t* test. (D) Representative images of ACE2 IHC in non-bronchial cells of murine lungs from male (intact, castrated, castrated + testosterone) and female (intact, intact + testosterone). (E) Quantitative analysis of ACE2 IHC in non-bronchial cells of murine lungs from indicated groups. *P* values were calculated by unpaired *t* test. (F) Co-expression of *Tmprss2* and *Ar* ISH in murine non-bronchial cells. (G) Co-expression of *Tmprss2* and *Sftpb* ISH in murine bronchial cells. (H) Co-expression of *Tmprss2* and *Sftpb* ISH in murine non-bronchial cells.

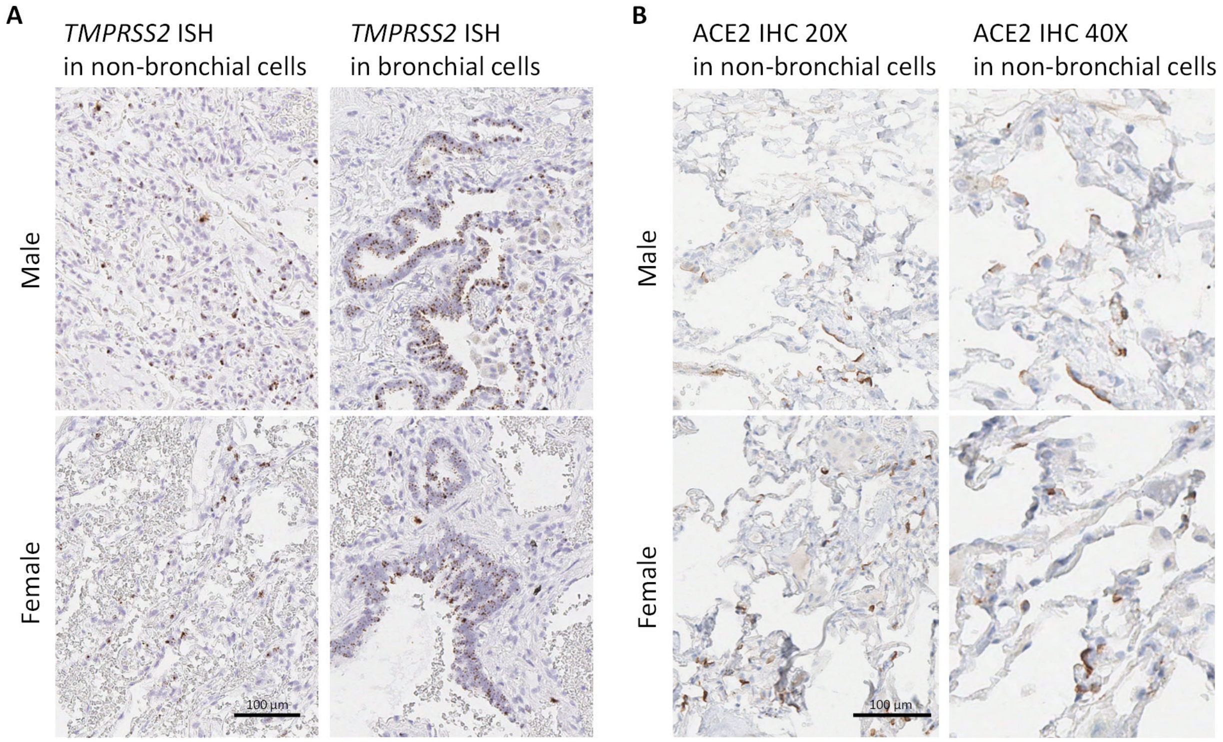

Figure S6. Expression patterns of *TMPRSS2* and ACE2 in human lungs. (A) Representative images of *TMPRSS2* ISH in bronchial and non-bronchial cells of male and female. (B) Representative images of ACE2 IHC in non-bronchial cells of male and female.

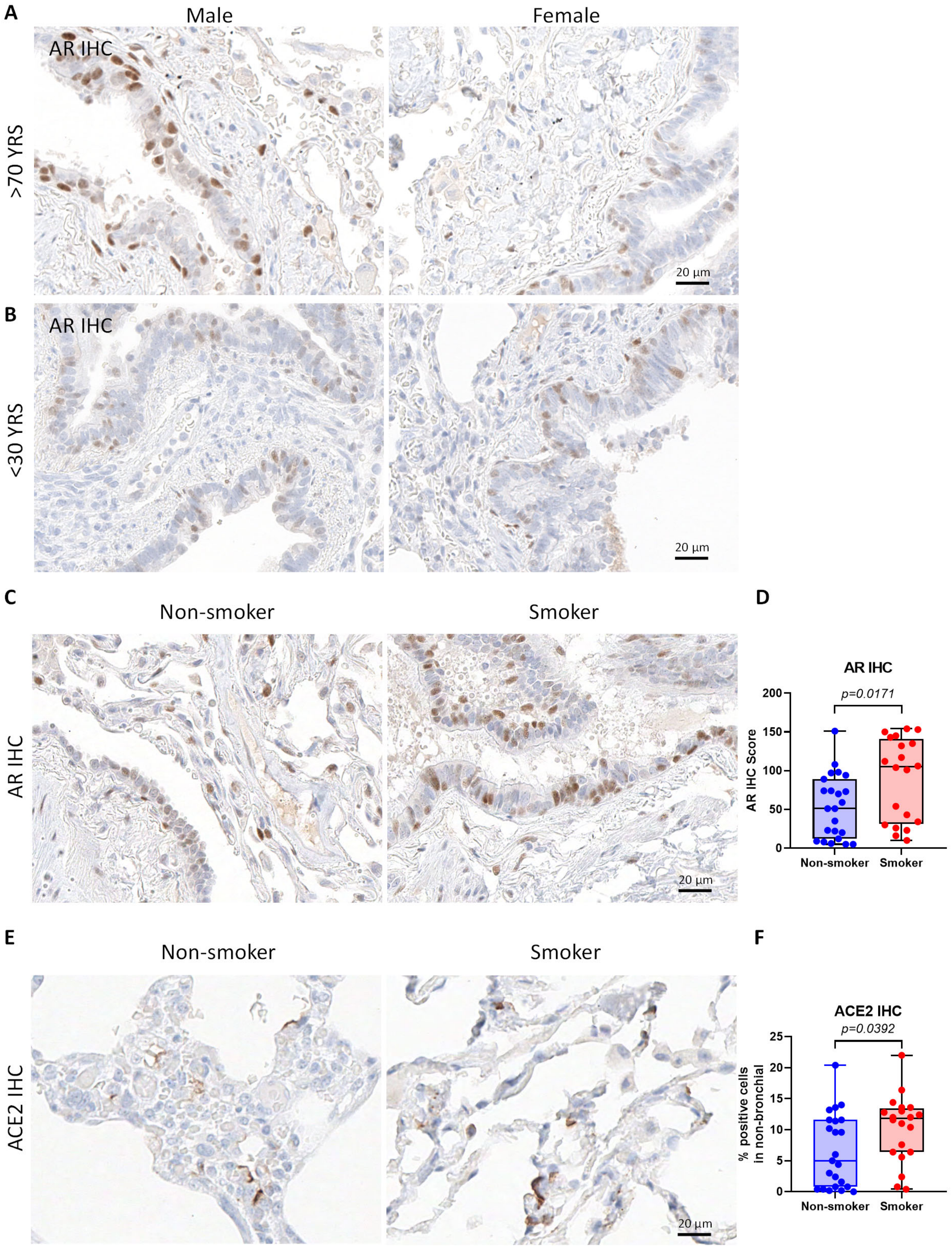
A
Male
Female
AR IHC
>70 YRS
20 µm
B
AR IHC
<30 YRS
20 µm
C
Non-smoker
Smoker
AR IHC
20 µm
D
AR IHC
p=0.0171
AR IHC Score
Non-smoker
Smoker
E
Non-smoker
Smoker
ACE2 IHC
20 µm
F
ACE2 IHC
p=0.0392
% positive cells in non-bronchial
Non-smoker
Smoker

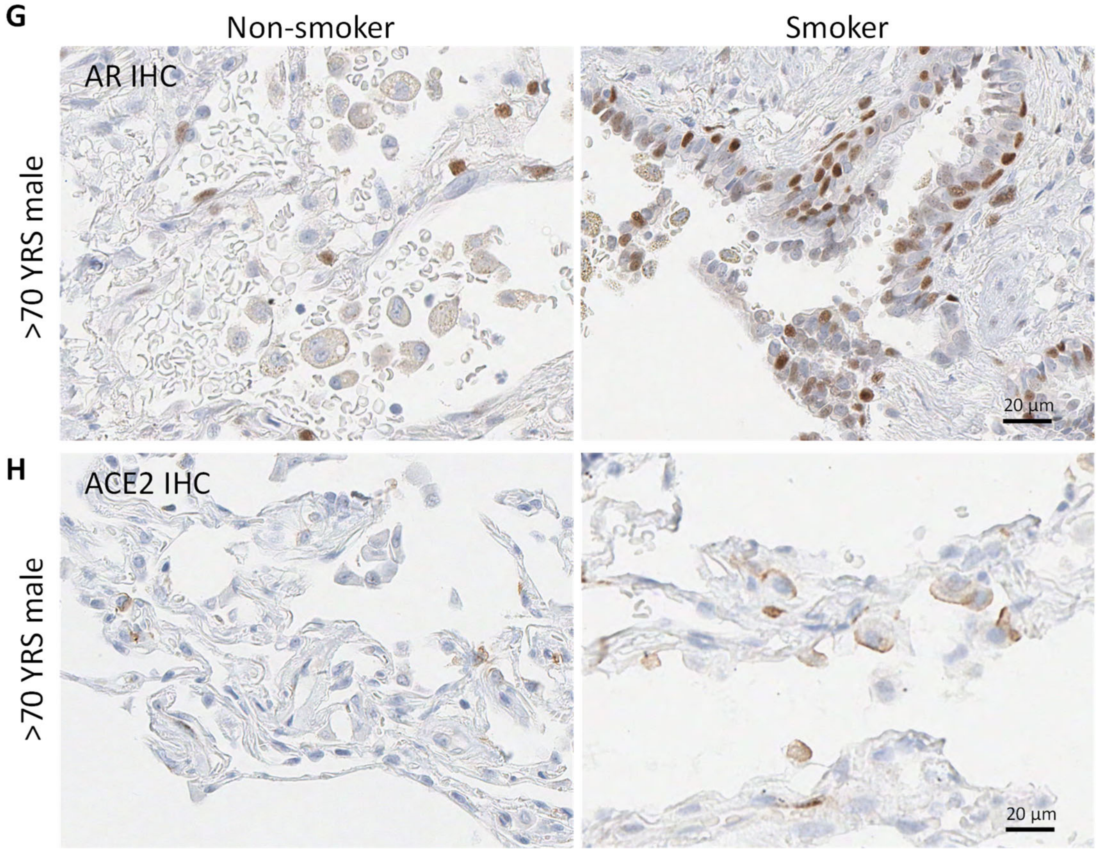

Figure S7. Smoking elevates AR and ACE2 expression in aged male lungs. (A) Representative images of AR IHC of human lungs in males and females over 70 years of age. Magnified images from the same field of view are shown in Figure 4A. (B) Representative images of AR IHC of human lungs in males and females under 30 years old. Magnified images from the same field of view are shown in Figure 4C. (C) Representative images of AR IHC in human lungs of non-smoker and smoker. (D) Quantitative analysis of AR IHC score in human lungs of non-smokers (n=23) and smokers (n=20). *P* values were calculated by unpaired *t* test. (E) Representative images of ACE2 IHC in human lungs of non-smoker and smoker. (F) Quantitative analysis of ACE2 IHC in human lungs of non-smokers (n=23) and smokers (n=20). *P* values were calculated by unpaired *t* test. (G) Representative images of AR IHC of aged male (>70 years old) lungs with different smoking statuses. Magnified images from the same field of view are shown in Figure 4E. (H) Representative images of ACE2 IHC of aged male (>70 years old) lungs with different smoking statuses. Magnified images from the same field of view are shown in Figure 4G.

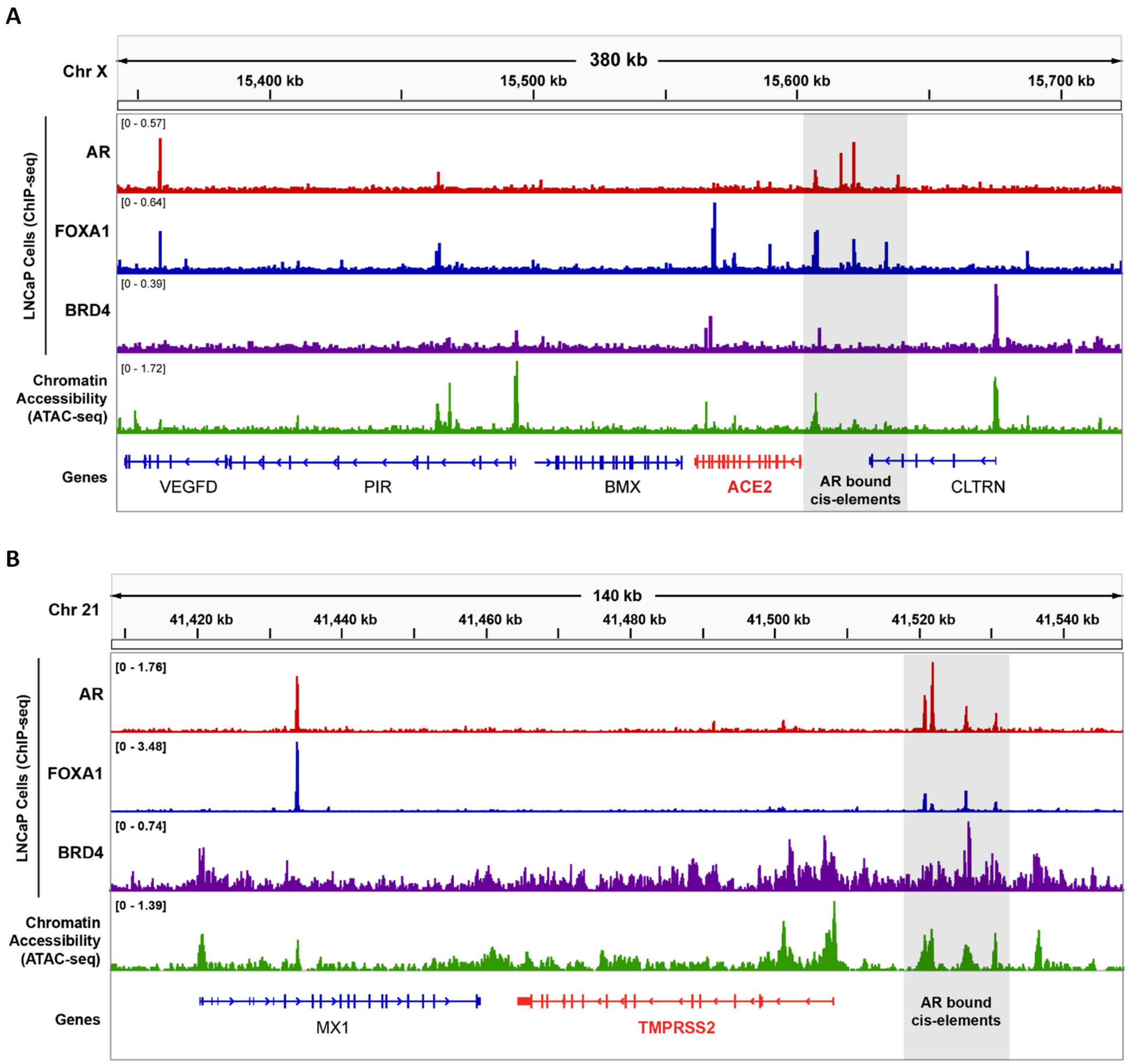

Figure S8. Androgen receptor protein binds to cis-elements of *ACE2* and *TMPRSS2*. (A) Genome browser representation of AR, FOXA1, and BRD4 binding events in comparison with chromatin accessibility on cis-elements of *ACE2* gene. (B) Genome browser representation of AR, FOXA1, and BRD4 binding events in comparison with chromatin accessibility on cis-elements of AR-regulated *TMPRSS2* gene.

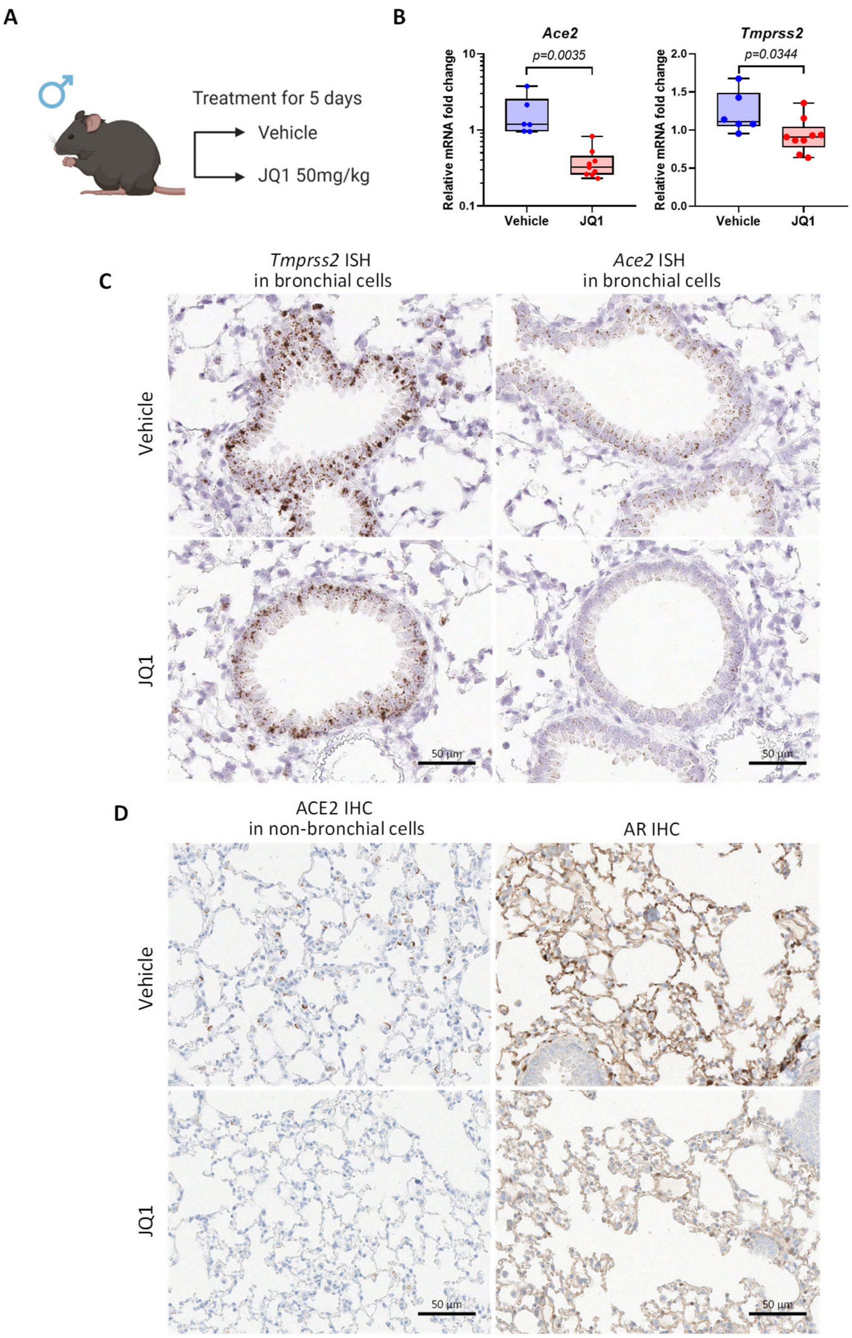

Figure S9. Bromodomain antagonist JQ1 decreases *Ace2* and *Tmprss2* expression in male murine lungs *in vivo*. (A) Murine study design of C57BL/6 male mice for vehicle (n=6) and 50 mg/kg JQ1 (n=9) treatment for 5 consecutive days. (B) Relative mRNA levels of *Tmprss2* and *Ace2* of individual murine lungs in indicated groups. N=6 for vehicle group, and n=9 for JQ1 treated group. *P* values were calculated by unpaired *t* test. (C) Representative images of *Tmprss2* and *Ace2* ISH in bronchial cells in murine lungs from vehicle and JQ1 treated male mice. Magnified images from the same field of view are shown in Figure 6A. (D) Representative images of ACE2 IHC in non-bronchial cells and AR IHC in murine lungs from vehicle and JQ1 treated male mice. Magnified images from the same field of view are shown in Figure 6A.

SUPPLEMENTARY METHODS

Immunohistochemistry (IHC) scoring

For AR, the final product score was calculated by multiplying the percentage of immune-positive cells by staining intensity (based on the following criteria: low intensity = score 1, moderate intensity = score 2, high intensity = score 3) with a possible maximum score of 300. For ACE2, the total number of immune-positive cells out of 500 (n/500) in the representative non-bronchial regions (alveolar predominantly) was recorded and expressed as a percentage of positive cells for quantification purposes.

RNA *in situ* hybridization (RNA-ISH) probes and scoring

Target probes used in RNA-ISH and catalog numbers in parentheses are as follows: Hs-*TMPRSS2* (470341), Hs-*ACE2* (848151), Hs-*AR* (400491), Mm-*Tmprss2* (496721), Mm-*Ace2* (417081), Mm-*Ar* (316991), Hs-*TMPRSS2*-C2 (470341-C2), Mm-*Tmprss2*-C2 (496721-C2), Hs-*SFTPB*-C2 (544251-C2), Mm-*Sftpb*-No-XHs-C2 (539421-C2).

For scoring, the number of dots per cell was counted and expression level was evaluated according to the RNAscope scoring criteria as follows: score 0 = no staining or <1 dot per 10 cells, score 1 = 1-3 dots per cell, score 2 = 4-9 dots per cell and none or very few dot clusters, score 3 = 10-15 dots per cell and <10% dots in clusters, score 4 = >15 dots per cell and > 10% dots are in clusters. The H-score was calculated for each examined tissue section as the sum of the percentage of cells with score 0-4 [(A%x0)+(B%x1)+(C%x2)+(D%x3)+(E%x4), A+B+C+D+E=100], using previously published scoring criteria (8, 9).

Immunoblotting

Cell lysates were harvested in Pierce radioimmunoprecipitation assay (RIPA) buffer (Thermo Scientific) containing protease (Roche) and phosphatase (Millipore) inhibitor cocktails. Protein concentration was measured using the Detergent Compatible (DC) Protein Assay (Bio-Rad). Denatured lysates were separated on NuPage 4-12% Bis-Tris Midi Protein gels (Novex) and transferred to 0.45 μm polyvinylidene difluoride membrane (Immobilon) using a TransBlot Turbo dry transfer machine (Bio-Rad). The membrane was incubated in blocking buffer (5% non-fat dry milk, Tris-buffered saline with 0.1% Tween-20) for one hour at room temperature. The membrane was then incubated with primary antibody for one hour at room temperature, followed by overnight incubation at 4°C. Chemiluminescent detection using ECL Prime (Amersham) and signal were visualized by an Odyssey imaging system (Li-Cor). Primary antibodies were ACE2 (Cat No. MA5-32307, Invitrogen), AR (Cat No. sc-7305, Santa Cruz), tubulin (Cat No. 66031, Proteintech), actin (Cat No. AC026, ABclonal), and GAPDH (Cat No. 3683S, Cell Signaling). All antibodies were used at dilutions suggested by the manufacturers.

RNA isolation and quantitative real-time PCR (qPCR)

Total RNA was extracted from cells or tissue using the miRNeasy mini kit (Qiagen), and complementary DNA (cDNA) was synthesized from 1 μg total RNA using the High Capacity cDNA Reverse Transcription Kit (Applied Biosystems). qPCR was performed using either fast SYBR green or TaqMan master mix (Applied Biosystems) on the ViiA7 Real-Time PCR System (Applied Biosystems). Target mRNA expression was quantified using the ΔΔCt method and normalized to *GAPDH*. Primer sequences used for SYBR green qPCR were:

TMPRSS2-Forward (CCTCTAACTGGTGTGATGGCGT)

TMPRSS2-Reverse (TGCCAGGACTTCCTCTGAGATG)

ACE2-Forward (TCCATTGGTCTTCTGTCACCCG)

ACE2-Reverse (AGACCATCCACCTCCACTTCTC)

GAPDH-Forward (GTCTCCTCTGACTTCAACAGCG)

GAPDH-Reverse (ACCACCCTGTTGCTGTAGCCAA)

Tmprss2-Forward (AAGTCCTCAGGAGCACTGTGCA)

Tmprss2-Reverse (CAGAACCTCCAAAGCAAGACAGC)

Ace2-Forward (TCCATTGGTCTTCTGCCATCCG)

Ace2-Reverse (AGACCATCCACCTCCACTTCTC)

Gapdh-Forward (CATCACTGCCACCCAGAAGACTG)

Gapdh-Reverse (ATGCCAGTGAGCTTCCCGTTCAG)

Probes used for TaqMan qPCR were: *TMPRSS2* (Hs01122322_m1); *ACE2* (Hs01085333_m1); *AR* (Hs00171172_m1); *ACTIN* (Hs01060665_g1); *Tmprss2* (Mm00443687_m1); *Ace2* (Mm01159006_m1); *Gapdh* (Mm99999915_g1).

Castration and testosterone treatments in mice

Eight-week old wild-type C57BL/6 mice were purchased from the Jackson Laboratory. 18 male mice were divided into three groups (group I for intact, group II for castration, group III for castration followed by testosterone (T) treatment), and 12 age-matched female mice were divided into two groups (group IV for intact, group V for testosterone treatment). Seven days after castration of group II/III, group III of male mice and group V of female mice received androgen for an additional five days (1 mg testosterone/mouse/day in 0.1 mL of corn oil with 5% dimethyl sulfoxide (DMSO) by subcutaneous injection, corresponding to 30 μg T/g of body weight),

whereas the control group (group I/II/IV) received vehicle. Animals were killed on day six of testosterone treatment, and the lungs were inflated by 1 ml (phosphate-buffered saline) PBS through the trachea. Left lung lobes were fixed by neutral formalin solution for formalin-fixed paraffin-embedded (FFPE) samples and subsequent IHC analysis. Right lung lobes were flash frozen for RNA and protein isolation. All experiments were performed according to the guidelines for animal experiments at the Shanghai Institute of Biochemistry and Cell Biology, Chinese Academy of Sciences.

Mass spectrometry for serum testosterone detection

A liquid chromatography-mass spectrometry (LC-MS) method was established to detect testosterone. Samples were analyzed on a HPLC station (Agilent) with G4204A pumps/G1367E auto-sampler/G1316A column oven/triple quadrupole 6490 (Agilent) equipped with an electrospray ionization source. The gradient mobile phase (0.3 mL/min) was a mixture of water (A) and methanol (B) containing 0.1% formic acid: 0 minutes 45% B, 9 minutes 54% B, 9.51 minutes 90% B, 12.5 minutes 90% B, 12.51 minutes 45% B, and kept at 45% B until the end of the run (15 minutes). Androgen was ionized with drying gas (14 L/min) at 200 ºC. The nebulizer pressure was 20 psi and the capillary voltage was 3,000 V (negative and positive). Methanol and water were LC/MS grade (Fisher Scientific).

JQ1 treatment in mice

Eight-week old male C57BL/6 mice were purchased from the Jackson Laboratory. Six mice were treated with vehicle [10% HPβCD (hydroxypropyl beta cyclodextrin) in D5W], and nine mice were treated with 50 mg/kg JQ1 daily for 5 days by intraperitoneal injection. JQ1 (Cat. 11187, Caymen) was first dissolved in DMSO and then diluted in 10% HPβCD daily prior to injection.

Mice were killed four hours post-last dosing, and lungs were inflated by PBS before tissue collection. Left lungs were fixed for FFPE, and right lungs were snap frozen for RNA extraction. The University of Michigan (U-M) Institutional Animal Care and Use Committee (IACUC) approved this study.

Murine lung preparation for single cell nuclei sequencing

Eight-week old male C57BL/6 mice were purchased from the Jackson Laboratory. Four mice were surgically castrated and monitored for post-surgical health condition. Twelve days post-castration, four castrated male mice, together with four age-matched intact male mice, were killed and lungs perfused through the right ventricle with 10-20 ml PBS. After perfusion, the entire lung was washed twice with PBS and flash frozen in liquid nitrogen for use immediately or stored at -80ºC for single nuclei isolation. The U-M IACUC approved this study.

Single nucleus RNA sequencing

All buffers were pre-chilled to 4ºC, and all steps were performed on ice or at 4ºC, unless otherwise mentioned. Flash-frozen mouse lung tissues were minced with a fresh, RNase-free scalpel and added to a tube containing chilled nuclei isolation buffer (10 mM Tris-HCl, pH 7.5, 10 mM NaCl, 3 mM $MgCl_2$, 0.16 % Nonidet™ P40, 0.2 U/µL RNase inhibitor) at a tissue:buffer ratio of 0.1g:100 µL. After incubating at 4ºC with rotation for 10 minutes, tubes were incubated at 4ºC for two minutes to settle debris, and the supernatant containing the nuclei was collected. The supernatant was centrifuged at 500g, 4ºC for five minutes to pellet the nuclei. The supernatant was discarded and the pellet was resuspended in 500 µL resuspension buffer (1x PBS, 0.4 % bovine serum albumin (BSA), 0.2 U/µl RNase inhibitor) and passed through a 70 µm filter. The above step was

repeated twice with a 40 µm filter. The nuclei suspension was centrifuged at 500g, 4ºC for five minutes to pellet the nuclei, and the nuclei were resuspended in 100 µL of resuspension buffer. After counting (trypan blue staining), the nuclei suspension was diluted to a concentration of 1 million nuclei/mL and immediately used for 10x barcoding. Approximately 5,000 nuclei from each sample were captured in Gel Bead-In-EMulsions (GEMs) containing ~750,000 unique molecular indexes (UMIs) using the 10X Chromium Controller module. The GEMs were generated in a chip, containing Master Mix/cells mixture, Gel Beads, and partitioning oil (placed in distinct compartments). Subsequent to dissolution of the Single Cell 3' Gel Bead in a GEM, primers containing an Illumina P7 and R2 sequence (read 2 sequencing primer), a 14 bp 10x barcode, a 10 bp randomer, and a poly-dT sequence were released and mixed with cell lysate and reaction master mix. Reverse transcription produced barcoded, full-length cDNA. After GEMs were broken and cDNA cleaned using Solid Phase Reversible Immobilization beads, the contents were subject to PCR. The sheared (Covaris) amplified cDNA were subjected to end repair, A-tailing, adaptor ligation, and PCR. Samples were sequenced on an Illumina HiSeq.

Data processing and analysis for single cell/nuclei sequencing

Read alignment and quantification were conducted with Cellranger (3.1.0) and pre-mRNA reference built from mm10-2.1.0 with mkref function. Cells with extreme low and high numbers of UMI or detected genes (3 times median-absolute-deviations away from median), which were possible low-quality cells and doublets, were identified using the outlier function from Scater (10) and removed from further analysis. The downstream analysis was performed with Seurat v3 (11). Specifically, data were normalized by sequencing depth and log-transformed, followed by identification of high variable genes. Libraries prepared from multiple batches were corrected for

batch effects with integrate data function before dimension reduction with t-distributed stochastic neighbor embedding (t-SNE) (12). Cells were assigned to clusters using FindClusters function with resolution = 0.5. Known markers for major lung cell types were used to annotate identified cell clusters.

Analysis of expression levels of lung and prostate

To compare *ACE2*, *TMPRSS2*, and *AR* expression levels in lung and prostate, the mean expression of stably expressed genes (SEGs) across cell types and tissues were used as internal reference for each cell. SEGs were based on published gene lists which derived from early human and mouse development scRNA-seq datasets and proved to be more stable than commonly used housekeeping genes at the single cell level (13). To avoid using genes exhibiting low expression and low stability in the specific datasets we used, genes with SEG index estimated by scMerge (13) lower than the median were removed. Relative expression of *ACE2*, *TMPRSS2*, and *AR* was calculated by subtracting mean expression of selected stably expressed genes. All calculations were based on log-normalized UMI by Seurat.

SUPPLEMENTARY INFORMATION REFERENCES

1. J. C. Kimmel *et al.*, Murine single-cell RNA-seq reveals cell-identity- and tissue-specific trajectories of aging. *Genome Res* **29**, 2088-2103 (2019).
2. K. J. Travaglini *et al.*, A molecular cell atlas of the human lung from single cell RNA sequencing. *BioRxiv* doi:10.1101/742320 (2020).
3. A. C. Habermann *et al.*, Single-cell RNA sequencing reveals profibrotic roles of distinct epithelial and mesenchymal lineages in pulmonary fibrosis. *Sci Adv* **6**, 1-15 (2020).
4. M. S. B. Raredon *et al.*, Single-cell connectomic analysis of adult mammalian lungs. *Sci Adv* **5**, eaaw3851 (2019).
5. P. A. Reyfman *et al.*, Single-cell transcriptomic analysis of human lung provides insights into the pathobiology of pulmonary fibrosis. *Am J Respir Crit Care Med* **199**, 1517-1536 (2019).
6. G. H. Henry *et al.*, A cellular anatomy of the normal adult human prostate and prostatic urethra. *Cell Rep* **25**, 3530-3542 (2018).
7. W. R. Karthaus *et al.*, Regenerative potential of prostate luminal cells revealed by single-cell analysis. *Science* **368**, 497-505 (2020).
8. S. L. Skala *et al.*, Next-generation RNA Sequencing-based Biomarker Characterization of Chromophobe Renal Cell Carcinoma and Related Oncocytic Neoplasms. *Eur Urol* **78**, 63-74 (2020).
9. L. Wang *et al.*, VSTM2A Overexpression Is a Sensitive and Specific Biomarker for Mucinous Tubular and Spindle Cell Carcinoma (MTSCC) of the Kidney. *Am J Surg Pathol* **42**, 1571-1584 (2018).
10. D. J. McCarthy, K. R. Campbell, A. T. Lun, Q. F. Wills, Scater: pre-processing, quality control, normalization and visualization of single-cell RNA-seq data in R. *Bioinformatics* **33**, 1179-1186 (2017).
11. T. Stuart *et al.*, Comprehensive integration of single-cell data. *Cell* **177**, 1888-1902 (2019).
12. L. van der Maaten, G. Hinton, Visualizing Data using t-SNE. *J Mach Learn Res* **9**, 2579-2605 (2008).
13. Y. X. Lin *et al.*, Evaluating stably expressed genes in single cells. *Gigascience* **8** (2019).
